# Leprosy at the edge of Europe—Biomolecular, isotopic and osteoarchaeological findings from medieval Ireland

**DOI:** 10.1371/journal.pone.0209495

**Published:** 2018-12-26

**Authors:** G. Michael Taylor, Eileen M. Murphy, Tom A. Mendum, Alistair W. G. Pike, Bethan Linscott, Huihai Wu, Justin O’Grady, Hollian Richardson, Edmond O’Donovan, Carmelita Troy, Graham R. Stewart

**Affiliations:** 1 Department of Microbial Sciences, Faculty of Health and Medical Sciences, University of Surrey, Guildford, United Kingdom; 2 Archaeology and Palaeoecology, School of Natural and Built Environment, Queen’s University Belfast, Belfast, Northern Ireland; 3 Department of Archaeology, University of Southampton, Highfield Road, Southampton, United Kingdom; 4 Faculty of Health and Medical Sciences, University of Surrey, Guildford, United Kingdom; 5 Norwich Medical School, University of East Anglia, Norwich, England, United Kingdom; 6 Edmond O’Donovan & Associates, Archaeological Consultant, Bray, Co. Wicklow, Republic of Ireland; 7 Rubicon Heritage Services Ltd, Unit 2, Europa Enterprise Park, Midleton, Co. Cork, Republic of Ireland; University of Florence, ITALY

## Abstract

Relatively little is known of leprosy in Medieval Ireland; as an island located at the far west of Europe it has the potential to provide interesting insights in relation to the historical epidemiology of the disease. To this end the study focuses on five cases of probable leprosy identified in human skeletal remains excavated from inhumation burials. Three of the individuals derived from the cemetery of St Michael Le Pole, Golden Lane, Dublin, while single examples were also identified from Ardreigh, Co. Kildare, and St Patrick’s Church, Armoy, Co. Antrim. The individuals were radiocarbon dated and examined biomolecularly for evidence of either of the causative pathogens, *M*. *leprae* or *M*. *lepromatosis*. Oxygen and strontium isotopes were measured in tooth enamel and rib samples to determine where the individuals had spent their formative years and to ascertain if they had undertaken any recent migrations. We detected *M*. *leprae* DNA in the three Golden Lane cases but not in the probable cases from either Ardreigh Co. Kildare or Armoy, Co. Antrim. *M*. *lepromatosis* was not detected in any of the burals. DNA preservation was sufficiently robust to allow genotyping of *M*. *leprae* strains in two of the Golden Lane burials, SkCXCV (12-13^th^ century) and SkCCXXX (11-13^th^ century). These strains were found to belong on different lineages of the *M*. *leprae* phylogenetic tree, namely branches 3 and 2 respectively. Whole genome sequencing was also attempted on these two isolates with a view to gaining further information but poor genome coverage precluded phylogenetic analysis. Data from the biomolecular study was combined with osteological, isotopic and radiocarbon dating to provide a comprehensive and multidisciplinary study of the Irish cases. Strontium and oxygen isotopic analysis indicate that two of the individuals from Golden Lane (SkCXLVIII (10-11^th^ century) and SkCXCV) were of Scandinavian origin, while SkCCXXX may have spent his childhood in the north of Ireland or central Britain. We propose that the Vikings were responsible for introducing leprosy to Ireland. This work adds to our knowledge of the likely origins of leprosy in Medieval Ireland and will hopefully stimulate further research into the history and spread of this ancient disease across the world.

## Introduction

Leprosy is a chronic granulomatous disease caused by *M*. *leprae* and the recently identified *M*. *lepromatosis* [1]. Susceptible individuals may be afflicted along a spectrum of severity depending on immune system status, with highest resistance occurring in tuberculoid leprosy and diminishing through the borderline form of disease to lepromatous leprosy (LL) that manifests in those with lowest resistance. A recent WHO classification recognizes two simplified categories of either paucibacillary or multibacillary forms of the disease [2] to essentially assist with treatment regimes. Skeletons from the archaeological record with characteristic lesions [3,4] represent individuals at the multibacillary end of the disease spectrum. Leprosy has probably been in the neighboring island of Britain since Roman times [5] and increased steadily throughout the Anglo-Saxon period. Early cases are known from Great Chesterford in Essex [6], dating to the 5^th^-6^th^ centuries AD and Hoxne in Suffolk (9^th^-11^th^ century AD) [7]. To date, biomolecular studies have tested only for *M*. *leprae*, which does appear to have been the species underlying the rise in European leprosy, but the involvement of *M*. *lepromatosis* (if any) remains to be elucidated.

By the Middle Ages, leprosy was an endemic disease in Europe and one that elicited a complex social and theological response, largely related to a fear of catching the disease. The theological response appears to have been entwined with the concept of purgatory and the harsh perspective that the disease was a physical manifestation of immorality, a concept that was modified in the 11^th^ century when the leprosy sufferer was sometimes viewed as one who endured purgatory on earth and would be permitted to pass straight to heaven after death [8].

A range of secondary evidence for the disease exists in Ireland, including numerous references in historical sources, such as various annals and religious texts. A range of place names (e.g. Baile-na-lobhar, “Town of the Lepers”, now Leopardstown; Knocknalower in Mayo and Cluain-na-lobhar in Kerry, both meaning “Pasture of the Lepers” and Poulnalowr in Cork, meaning “Pool of the Lepers”) and the occurrence of leper squints or hagioscopes (e.g. in St Nicholas, Carrickfergus and St Mary’s Cathedral, Limerick) are also suggestive of a Medieval presence of the disease on the island. Furthermore, at least fifty hospitals with apparent associations with leprosy existed in Medieval Ireland, the majority of which were located in Munster and Leinster [9]. Hospitals in Dublin associated with the disease include those dedicated to St James, St John the Baptist and St Stephen, all of which were founded in the 13^th^ century [9,10].

Despite this widespread secondary evidence, only three archaeological sites have produced osteoarchaeological cases of leprosy in Medieval human remains; a single case (Sk171) from St Patrick’s Church, Armoy, Co. Antrim [10], three cases (SkCXLVIII, SkCXCV and SkCCXXX) from the cemetery of St Michael Le Pole, Dublin [11,12] and an unpublished example (Sk1494) from Ardreigh burial ground, Co. Kildare [13].

Ireland is of particular interest in the history of leprosy as it was never part of the Roman world nor underwent any significant occupation by later Anglo-Saxon settlers, although a handful of possible Anglo-Saxon burials have been discovered [14]. Ireland’s island nature does not seem to have been a barrier to the movement of people during early Medieval times and there is ample historical evidence that Irish religious scholars, such as Columbanus (543–615 AD), for example, travelled throughout Europe [15]. Furthermore, a recent review of exotic objects has indicated three main phases of trade in early Medieval Ireland dominated by particular connections—Mediterranean (c. AD 400–600), Gaulo-Frankish (c. AD 600–800) and Hiberno-Scandinavian (c. AD 800–1150) [16]. The Viking connections had a particularly prominent impact on the island through the establishment of towns and include the discovery of Viking burials, particularly in the center of Medieval Dublin, which is an indication of the significance of this area during the ninth and tenth centuries [17]. Indeed, four Viking burials were identified just outside the boundary of the graveyard of St Michael le Pole, one of the sites included in the current study. One of these individuals was dated to AD 678–832, thereby indicating that the individual pre-dated the known establishment of the Viking longphort in AD 841 [11].

The next major incursion of newcomers occurred in the late AD 1160s, when the first of the Anglo-Norman incursions commenced, with attacks on the ports of the southeast, including Dublin, in AD 1170 [15]. This military activity resulted in much of the south and east of Ireland coming under Anglo-Norman control, particularly the colonial hinterland of Dublin (the Pale). This area remained under the control of English kings until the emergence of an Anglo-Irish identity in the wake of the major outbreak of plague in the mid-fourteenth century [18]. Ireland was evidently well connected throughout the Medieval period and we speculated that the lineage(s) of Irish leprosy may reflect one or more of these earlier connections, particularly those known to have involved larger-scale population movement, such as the Norse or later Anglo-Norman incursions.

Understanding of the evolution and spread of leprosy has advanced in recent years, largely through studies that have examined the *M*. *leprae* genome, both geographically and temporally [19–22]. The present study aims to place Ireland within this growing framework of research through the analysis of ancient strains of *M*. *leprae* in Irish cases. This is combined with palaeopathological and isotopic analysis, together with radiocarbon dating to understand the likely origins and development of the disease in Ireland. We have also looked for evidence of the second pathogen, *M*. *lepromatosis*, given its identification in red squirrels in neighboring Scotland and their possible zoonotic role in the spread of the disease [23,24].

## Irish medieval leprosy—The evidence

**Fig 1:**
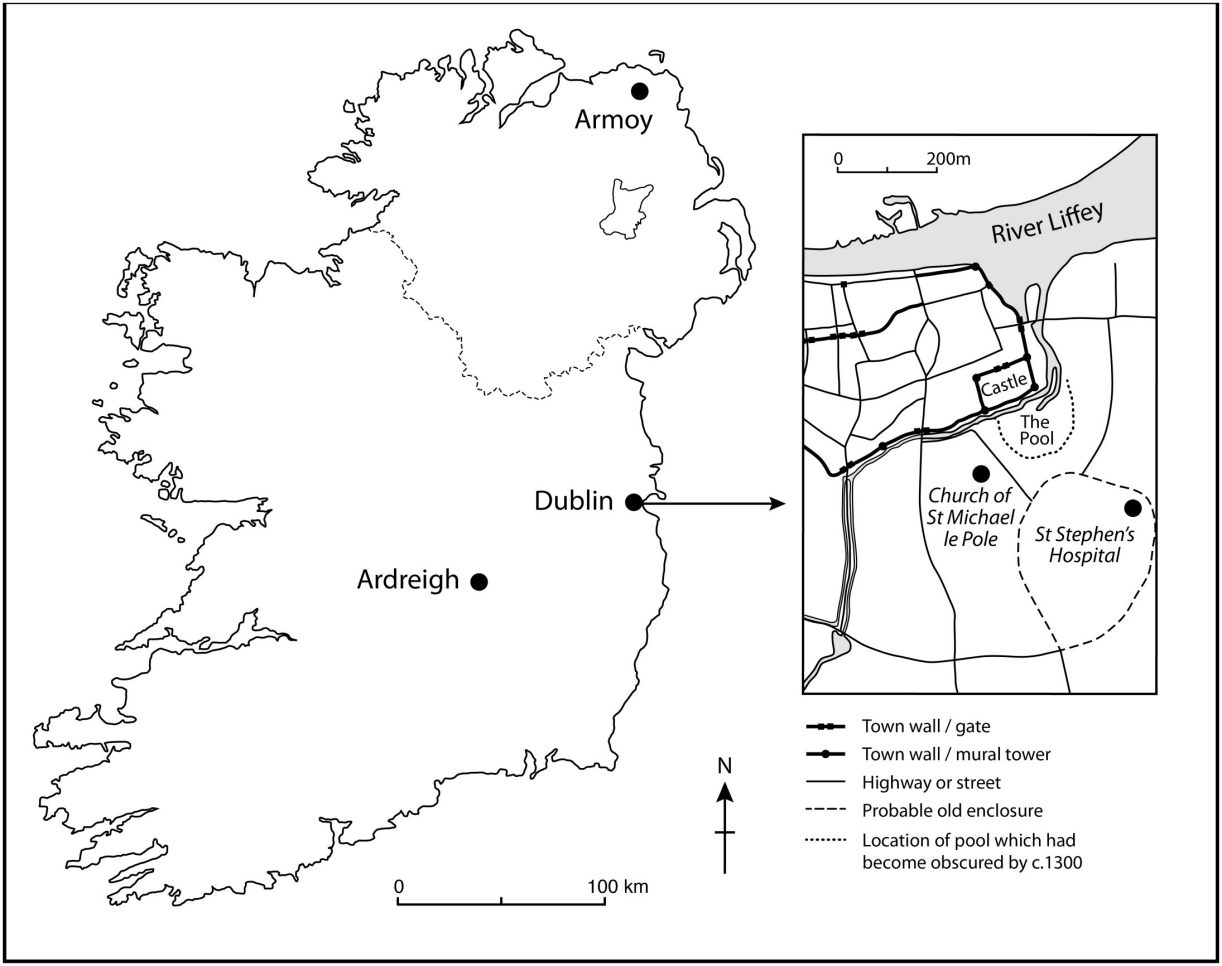
Map showing the location of the sites included in the study. The insert depicts features apparent in the vicinity of the Golden Lane site near the church of St Michael Le Pole, including St Stephen’s leprosarium, c. AD 1300. The original maps upon which the insert is based are fully available under a CC BY license, with permission from Royal Irish Academy, original copyright 2016 [25].

The study focuses on five cases of probable leprosy identified in human skeletal remains excavated from inhumation burials. Three of the individuals derived from the cemetery of St Michael Le Pole, Golden Lane, Dublin, while single examples were also identified from Ardreigh, Co. Kildare, and St Patrick’s Church, Armoy, Co. Antrim (Fig 1).

## Methods and materials

The Golden Lane cases (SkCXLVIII, SkCXCV and SkCCXXX) were excavated under licence number 04E1030 and Ardreigh Sk1494 under licence number 00E0156. No licence was required for the Armoy case (Sk171) as it was excavated in the jurisdication of Northern Ireland in 1997 before a licence numbering system was introduced. National Museum of Ireland export and destructive analysis licences applying in this study were No. 5651 for Golden Lane and No. 5923 for the Ardreigh case. No licence was required for the Armoy case. The Armoy case is currently stored in Queen’s University Belfast, while the Ardreigh and Golden Lane cases are curated by the National Museum of Ireland in Dublin. The remains can be accessed by contacting the relevant organization. All necessary permits were obtained for the study, which complied with all relevant regulations.

The Dublin skeletons originated from the graveyard associated with the church of St Michael le Pole at Golden Lane, where excavations took place in advance of development in 2005 [11,12]. The remains of 272 individuals were discovered, three of which displayed osteological lesions suggestive of leprosy. The church of St Michael le Pole is located 100m south of the walls of the Viking and Anglo-Norman town of Dublin. The church sits on a ridge overlooking the River Poddle that separates the church site from the city to the north and the Black Pool (*Dubh Linn* in Irish) to the east from which Dublin derives its name. It seems significant that the church of St Michael Le Pole is located around 300 m from the known 13^th^-century St Stephen’s leprosarium (see Fig 1). The individual from Armoy was discovered in 1997 during excavations in the interior of St Patrick’s Church in Armoy, Co. Antrim, during its refurbishment, and was the only one of 56 individuals to display the lesions [10,26]. The skeleton from Ardreigh was one of 1,259 recovered from a cemetery near the town of Athy, Co. Kildare, during excavations undertaken in advance of a road realignment between 2000–2003 and 2007–2008. It was the only one considered to display leprosy [13]. Neither the sites at Armoy or Ardreigh have any known associations with leprosy.

### Osteology

The lesions apparent in the cases from Golden Lane [12] and Armoy have already been published [10, 26] and the details are included in the S1 File. The Ardreigh individual has not been previously published. A control individual with no osteological evidence of leprosy, SkCCL from Golden Lane, was also included in the study.

The state of preservation was assessed following the guidelines of McKinley [27]. Sex was determined on the basis of pelvic and cranial morphology following standard recommendations [28–31]. Juvenile age-at-death was estimated using a combination of epiphyseal fusion data, dental eruption and dental calcification [32,33]. Adult age-at-death determinations were based on the assessment of late fusing epiphyseal sites throughout the cranial and post-cranial skeleton [32], in addition to the assessment of the state of degeneration of the auricular surface of the ilium [34], pubic symphysis [35]and dental attrition [36]. Broad age categories were used and adults were identified as young adults (18–35 years), middle-aged adults (35–50 years) or older adults (50+ years) [30].

The pathological analysis involved a detailed morphological examination of the lesions, which were then documented and photographed. Leprosy is known to produce a distinct pattern of lesions with a focus on the rhino-maxillary area of the skull and the bones of the hands and feet. The facial lesions only occur in the lepromatous form of the disease whereas the post-cranial lesions can also arise in the other clinical forms of the condition [37]. The rhino-maxillary lesions are related to infection and inflammation and manifest as destruction of the anterior nasal spine, atrophy and recession of the maxillary alveolar bone adjacent to the incisors, which results in their subsequent loss, absorption of the nasal septum and conchae, remodelling of the margins of the nasal cavity and pitting or erosion of the palatine process of the maxilla [38,39]. The lesions of the hands and feet involve changes to the metacarpals, metatarsals and phalanges that arise from diaphyseal remodelling and acroosteolysis and eventually result in the bone having a ‘sucked candy’ appearance. Other characteristic lesions include ‘cup and peg’ deformity of the metatarsal/metacarpal-phalangeal and interphalangeal joints as well as the occurrence of dorsal tarsal exostoses and generalized signs of infection, including periostitis and bone necrosis [39,40]. Nerve damage can result in the bones of the hands becoming permanently flexed and pressure erosions may arise on the volar surfaces of the distal phalanges [4]. Nerve damage can also cause anaesthesia of the skin and this is particularly problematic for the feet since the individual becomes oblivious to injuries which are then at risk of secondary infection through the introduction of bacteria from external sources. As such, the occurrence of both leprous and non-leprous bacteria in the feet can result in the spread of infection to the lower limbs and septic arthritis and diffuse periostitis may be apparent [4].

### DNA extraction

Details of the bone samples taken for the DNA analysis can be found in the S2 File.

#### 1. At the University of Surrey (UoS)

Bone fragments were ground to a fine powder using sterilised pestles and mortars. The powders were weighed and divided into two amounts. One set was extracted immediately for leprosy screening, while the other was set aside for subsequent genotyping.

DNA was extracted using an in-house version of the Boom method [41]. In this procedure, 6M guanidinium thiocyanate (GUSCN, product G9020, US Biologicals, Salem, MA.) containing 1% Triton X-100 (Sigma-Aldrich, T8787) was dissolved in 1x Tris-EDTA buffer (Sigma-Aldrich, T9285) adjusted to pH 6.5 with 3M sodium acetate, pH 5.5 (Ambion, ^™^ product 9740). Bone powder was mixed with 1ml of the GUSCN buffer on a mixing wheel for 1hr at 4°C. The samples were then subjected to 3 freeze-thaw cycles to assist with DNA recovery. The bone powder was removed by centrifugation at 12,000 x r.p.m. and the supernatant buffer transferred to a fresh 1.5ml Eppendorf tube. Pre-washed silica suspension (40μl of 0.5–10μm, Sigma-Aldrich, S5631) was added and kept in contact for 3hrs to maximize recovery of fragmented DNA. After centrifugation, silica was further washed twice with 1ml aliquots of GUSCN extraction buffer, followed by 3 washes with 75% ethanol and finally with 1ml of acetone. After thorough drying of the silica pellet, DNA residues were eluted in 60μl HPLC grade water (Sigma-Aldrich) at 55°C. These were then sub-divided into 2 x 30 μl aliquots and stored in low retention plastic tubes to minimize loss of DNA through repeated freeze- thawing events. Extraction controls (less bone powder) were taken through the same procedure to check reagents were contamination free.

#### 2. At the University of East Anglia (UEA)

DNA was extracted from the bone powder received from UoS using a bead-beating approach. Initially, 700 μl bacterial lysis buffer (Roche UK- Cat. No. 4659180001) was added to the powder and vortexed before being transferred to a bead beating tube (Lysis Matrix B, 2ml tube (116911050, MP Biomedicals). The sample was bead-beaten for 2 minutes at full speed on a Tissue Lyser bead beater (Qiagen- Cat. No. 69980) and then pulse centrifuged (Eppendorf UK centrifuge model 5424). An aliquot (420 μl) was then transferred to a fresh Eppendorf tube and 20 μl of proteinase K (>600mAu/ml) (Qiagen, Cat. No. 19133) was added. The sample was then incubated at 65°C on a dry bath system (StarLab UK) for 10 minutes. Finally, DNA was purified on the MagNA Pure Compact machine (Roche UK) using the Roche MagnaPure Compact DNA_bacteria_V3_2 protocol (MagNA pure compact NA isolation kit I, Roche UK- product 03730964001).

#### *M*. *leprae* screening methods (at UoS)

Two separate PCR methods were used to screen for the RLEP element, present in multiple copies in the *M*. *leprae* genome. In the first of these, product formation was monitored using the intercalating dye EVA Green^™^ (Biotium). The second method employed a FAM labeled hybridization probe. Details of these two methods have been reported previously [42]. Finally, we also screened for the 18-kDa antigen locus (ML1795) using primers 18F 5‘-CTAATCGACTGTTGTTTGCGCAAC -3’ and 18R 5’- GCCAGCAACCGAAATGTTCGGA -3’ which amplify a 114 bp product. This was reported with a dual labeled hybridization probe, [JOE] 5’-CTGCGGTCAAAAGCCCGTCTTAGCCATG-3’[BHQ1] [42]. Experience shows that positivity for this single copy method is a useful indicator that SNP genotyping methods may be successful.

#### *M*. *leprae* screening methods (at UEA)

A SYBR green real-time PCR assay was used to detect the RLEP element and all PCR was performed on a Roche Lightcycler 480 Instrument II (Roche UK). PCR reactions (20μl) contained 10 μl of LightCycler^®^ 480 SYBR Green I Master mix (Roche, product 04707516001), 1 μl (500 nM) of each primer and 8 μl of DNA template. The sequences of the forward and reverse primer were F-5’-CATTTCTGCCGCTGGTATC-3’ and R-5’-ATCATCGATGCACTGTTCAC-3’. All PCR primers were purchased from Sigma-Aldrich. Real-time PCR cycling conditions were as follows: 1 cycle of 95°C for 5 minutes, 40 cycles of 95 °C for 30 seconds, 54 °C for 30 seconds, 72 °C for 30 seconds and an extension 72 °C for 5 minutes. A melt curve step of 95 °C for 5 seconds, 65 °C for 1 minute and a ramp rate of 0.11 °C/s to 97 °C with continuous fluorescence monitoring was used. The protocol included a final cooling step to 40 °C for 30 seconds.

#### *M*. *leprae* SNP genotyping methods (UoS only)

A series of PCR methods was used to genotype positive extracts. A number of these, used for characterizing *M*. *leprae* strains, have been previously published [42,43].

#### VNTR typing (UoS only)

Three variable number tandem repeat loci were chosen for variable number tandem repeat (VNTR) analysis. The details are shown in Table 1.

**Table 1:** VNTR PCR methods applied to positive Irish LL cases after Taylor and Donoghue, 2011 [4,4].

Locus	Genome location	Type	Locus ID	Repeat unit (bp)	Repeat motif	Amplicon size in TN (bp)
ML2344-ML2345	2785,364–2785,494	Pseudogene	(AGA)20	3	TTC	131
ML2172-ML2173	2583,816–2583,839	Hyp-pseudogene	(GTA)9	3	GTA	151
ML0058	72,683–73,686	*Esp e*	21–3	21	tgatcaacttgattcctggct	117
		pseudogene				

#### Screening for *M*. *lepromatosis* DNA (UoS)

Primers used were modifications of those described by Singh and colleagues [45]. The sequences of the pair used in the present study were F-5’-CTGTTCGTGAGGTACCGGTGAAA-3’ and R-5’-GTTCGGCCGGAGTGTAGGTGTTA-3’ These amplify a 135 bp fragment in the *hemN* gene, present in *M*. *lepromatosis* but absent from *M*. *leprae*. The PCR reagents and conditions were as described for *M*. *leprae* below, except that an annealing temperature of 58 °C was used.

#### Screening for *Mycobacterium tuberculosis* (MTB) complex DNA (UoS)

Extracts were also tested for the presence of *Mycobacterium tuberculosis* (MTB) complex organisms using a real-time PCR method for the IS*1081* repetitive element [46].

#### Human aDNA (UoS)—Mitochondrial DNA (mtDNA)

Two PCR methods were used to look for evidence of human mtDNA. Extracts were tested using primers which amplify a 116 bp region of the human mitochondrial DNA hypervariable region 1 (HVR-1). The sequences of these primers were: Forward (L15977-L15998) 5’-CCACCATTAGCACCCAAAGCTA-3’ and Reverse (H16092-H16070) 5’- ATACATAGCGGTTGTTGATGGGT-3’. Another variant of this PCR was used with an alternative reverse primer (H16255-H16236) with the sequence 5’-CTTTGGAGTTGCAGTTGATG-3’. In combination with the forward primer, this amplifies a product of 279 bp.

#### Human aDNA (UoS)—Amelogenin

Two separate gender-determining PCRs based on polymorphisms in the amelogenin gene were also applied. In the first method, males are identified by two PCR products, one of 105 bp from the Y chromosome (AMELY) and another of 290 bp from the X chromosome (AMALX), whereas females generate only one product of 290 bp. The sequences of the primers used in this procedure have been previously reported [47]. A second amelogenin method was also attempted [48]. This generates two bands from males of 106 bp and 112bp (AMELX and AMELY products respectively), and a single AMELX product of 106bp from females.

#### Human DNA screening (UEA)

A SYBR green real-time PCR assay was used to detect human mtDNA and all PCR was performed on the Roche Lightcycler 480 Instrument II. PCR reactions (20μl) contained 10 μl of LightCycler^®^ 480 SYBR Green I Master (Roche, 04707516001), 1 μl (500 nM) of each primer and 8 μl of DNA. The sequences of the forward (L15977-L15998) and reverse primer (H16092-H16070) were F-5’-CCACCATTAGCACCCAAAGCTA-3’ and R-5’- ATACATAGCGGTTGTTGATGGGT-3’. Real-time PCR cycling conditions were the following: 1 cycle of 95 °C for 5 minutes, 40 cycles: 95 °C for 30 seconds, 65 °C for 30 seconds, 72 °C for 30 seconds and an extension 72 °C for 5 minutes. A melt curve step of 95 °C for 5 seconds, 65 °C for 1 minute and a ramp rate of 0.11 °C/s to 97 °C with continuous fluorescence monitoring was used. A final cooling step to 40 °C for 30 seconds.

#### PCR amplification details (UoS)

PCR was performed in a final volume of 15μl, using a hot start Taq kit from Qiagen (product 203445). The reactions contained 25 pmol of forward and reverse primers, each in 1μl, 7.5 μl of the kit master mix, 1.5 μl non-acetylated bovine serum albumin (BSA, 10mg/ml, Sigma B4287) and 2μl of template. The kit magnesium ion concentration of 1.5 mM per reaction was supplemented to 2 mM for PCR methods using EVAGreen^™^ and to 3mM MgCl_2_ for real-time PCR with the RLEP and 18-kDa probes. The probes were used at a final concentration of 100 nM. Tube volumes were made up to 15 μl with molecular biology grade water (Sigma-Aldrich). After an initial activation step of 14 min at 95°C, 43 or 45 cycles of amplification were performed on an Mx3005P RT-PCR platform (Agilent Technologies).

The thermal profile of the amplification cycles consisted of denaturation at 95°C for 10s, annealing (range 52–60°C) for 30s and extension at 72°C for 30s. Fluorescence data was acquired during the extension step. Melt analyses was performed automatically at the end of runs monitored with EVAGreen^™^ and dissociation curves studied to identify likely positives.

#### Gel electrophoresis and automated DNA sequencing (at UoS)

PCR products were run out on 3% agarose gels in a TAE buffer system alongside appropriate DNA size markers (100 bp or 50 bp DNA ladders, Promega) to confirm product identity. For separation of the amelogenin amplicons (106/112bp), products were run on 4.5% MetaPhor^™^ agarose gels (Lonza Walkerville Inc.). In both instances, SYBR^™^ Safe DNA gel stain (Thermo Fisher Scientific) was used to visualize the bands. Positive samples for SNP or VNTR typing were bulk purified on 3% (wt/vol) low-melting-point agarose (Invitrogen); bands were excised and purified using a Geneclean DNA isolation kit (Cat.No.1001-200, mpbio.com). Templates were sequenced using both forward and reverse primers by Genewiz UK Ltd., Takeley, Essex, UK. The sequencing platform they used was the Life Technologies 3730xl DNA Analyzer, a 96 capillary instrument.

#### Visualisation of PCR products (UEA)

PCR products were visualised using an automated electrophoresis platform, 2200 TapeStation and Agilent software (Agilent Technologies-G2964AA). PCR products (1 μl) and loading buffer (10 μl) (Agilent Technologies- 5067–5366) were added to optical tube strips (Agilent Technologies-401428) and mixed before being placed into the TapeStation. The TapeStation then loaded the PCR products to the genomic screenTape (Agilent Technologies, 5067–5365) automatically. A DNA ladder (200 to >60,000 bp) (Agilent Technologies- 5067–5366) was run alongside the PCR products.

#### Sequencing of PCR amplicons (UEA)

Sanger sequencing of mtDNA and *M*. *leprae* PCR products was performed by Source Bioscience using the forward primer only (3.2pmol/μl) for the *M*. *leprae* and human PCR assays. The quantity of the DNA was determined before sending using the Qubit 3.0 Fluorometer (Thermo Fisher-Cat. No. Q33226) and the high sensitivity dsDNA assay kit (Thermo Fisher- Cat. No. Q32851).

#### Measures to prevent contamination (at UoS)

Measures to prevent contamination were adopted from the time of sampling onwards. Samples were taken from bone interiors which had not been handled. Separate laboratories were used for each of the three main stages of the aDNA analyses, these being extraction, amplification and post PCR analysis, such as gel electrophoresis and purification of products for sequencing. The pre- and post- PCR laboratories were physically separated by several hundred metres and independently equipped with personal protective equipment (PPE), pipettes, fridge-freezers, mixers and bench top centrifuges, disposable plasticware, filter tips and other reagents dedicated to the project.

Surfaces and equipment in contact with sample tubes (centrifuges, rotors, mixers, etc.) were cleaned before each assay. In the clean area, two control tubes, comprising reagents less bone powder, were taken through each extraction experiment to ensure reagents were contamination free before testing cases. Template blanks were run alongside bone extracts in the PCR machine to screen for random contamination. Positive controls were not included in any of the PCR experiments.

#### Measures to prevent contamination (at UEA)

Three separate laboratories were used for DNA extraction, PCR set up and post-PCR analysis in the Bob Champion Research and Educational Building. At the time this work was carried out, the laboratory was just under 2 years old and no previous aDNA or *M*. *leprae* DNA had ever been used in the laboratory. The laboratory used for DNA extraction was on a different floor from PCR set up and analysis. These rooms all were separately equipped with PPE, equipment and plastic ware. DNA extraction from bones samples took place in a class II microbiological safety cabinet which was thoroughly cleaned with 10% CHEMGENE HLD4L laboratory disinfectant (MediMark Scientific-XTM309) prior to use. All surfaces in the three separate labs were thoroughly cleaned with 2% CHEMGENE prior to use. Positive controls were not included in any of the PCR experiments. “No template” controls were run alongside bone extracts in all PCR runs to screen for contamination.

#### Whole genome amplification, enrichment and analysis (UoS)

DNA was extracted, enriched and assessed at the University of Surrey. The protocol has been fully described [20]. This involved removing uracil residues with USER enzyme (New England Biolabs, Hitchin, UK), repairing the DNA, ligating linkers to the ends and PCR amplifying before enriching for *M*. *leprae* sequences. Samples were pair-end sequenced with double indices on a HiSeq ^™^ X Ten platform (Genewiz Ltd., Takely, Essex, UK).

### Stable isotope analysis

The isotopic analysis was undertaken at the University of Southampton. Strontium and oxygen isotopic analysis were carried out on tooth enamel of the three Golden Lane cases to shed light on each individual’s childhood origins; no dental samples were available for either the Armoy or Ardreigh cases. Where available, samples of rib and long bone from each individual were sampled for carbon and nitrogen isotopic analysis for the purposes of dietary reconstruction. Details of the samples taken are provided in the S2 File.

#### Strontium

For strontium isotope analysis, the teeth sampled were permanent second or third molars. Longitudinal sections approximately 1mm thick of enamel and some incidental dentine were removed using a hand drill and diamond cutting disk. The samples represent a section of the complete length of the enamel from the crown to the cervix. Samples were cleaned in an ultrasonic bath in 18MΩ H_2_O for ten minutes and dried overnight in an oven at 60°C.

The samples were mounted in the laser cell by pressing into Blu-Tack. Strontium isotopic analysis was performed on a Finnegan Neptune multi collector ICP-MS with a New Wave 193 nm ArF homogenized excimer laser, using the oxide reduction technique of De Jong and colleagues [49,50]. The measurement of Sr isotopes by laser ablation has only recently been made reliable. The primary difficulty has been the molecular interference on ^87^Sr of ^40^Ca^31^P^16^O^+^, which is the primary constituent of the enamel matrix. Other potential problems come from double charged rare earth elements which give mass-to-charge ratios of between 84 and 88, calcium-calcium and calcium-argide dimers which can interfere with ^84^Sr, ^86^Sr and ^88^Sr, in addition to potential ^87^Rb and ^86^Kr interferences. Our approach has been to minimize oxide formation (monitored as ^254^(UO)^+^/^238^U^+^) through careful control of plasma conditions, and to monitor and reject teeth that have significant rare earth concentrations which we consider diagenetic. We correct for the ^86^Kr using an on-peak gas blank and for rubidium interference using the natural ^87^Rb/^85^Rb ratio of 0.385617. A small positive offset from known values ^87^Sr/^86^Sr of standards is usually observed but is within the precision of a typical measurement.

Time series of strontium isotopes are obtained as continuous data by moving the tooth along its growth axis (at 2.5 or 5μms^-1^) as the laser pulses with a repetition rate of 10Hz and spot size 110 μm, giving a fluence of c.8.6 Jcm^-2^. Repeat analysis of an in-house ashed bovine pellet standard (BP1) bracketing analyses of the teeth, showed an offset of +156±61 ppm (1 sigma) for the laser ablation analyses over TIMS values. This is within the precision of individual measurements of 200-600ppm and the total variation between the teeth of c. 5000ppm and is therefore considered insignificant to our interpretation of the isotopes.

#### Oxygen

For oxygen isotope analysis a second section was taken from each tooth. Surface dirt and any dentine was removed using a dental burr. The sample was ground to a fine powder in an agate mortar under acetone. The powder was leached for 10 minutes in 10% acetic acid to remove diagenetic carbonate then centrifuged and washed in 18MΩ H_2_O three times and dried. Two 5 μg aliquots were weighed for analysis.

Oxygen isotope analyses were performed on a Thermo Scientific Kiel IV Carbonate device coupled with a MAT253 isotope ratio mass spectrometer on c. 0.6mg samples. Samples were calibrated and drift corrected by bracketing with NBS18 and 19. Results are quoted as δ values relative to PDB. Quality control standards give reproducibilities at 1σ of ±0.01 for δ^13^C and ±0.05 for δ^18^O. For comparison with published measurements on enamel phosphate oxygen isotopes and rainwater δ18O, structural carbonate values were converted to phosphate values and then to drinking water equivalents [51,52].

#### Carbon and nitrogen

Trabecular bone turns over 3–10 times faster than cortical bone [53] so the isotopes from the ribs will represent the more recent average diet than from the femur samples. For C and N isotope analysis, circa 500 mg bone ‘chunks’ were demineralized over several days in 0.5M HCl at room temperature, centrifuged, rinsed and filtered. To remove contamination, the sample was treated with 0.1M NaOH for 30 minutes, rinsed and treated with 0.5M HCl for 15 minutes and further rinsed. The residue was gelatinized in pH 3 HCl at 70 °C for 24 hours. Insoluble residue was then removed by centrifuging and filtering, and the soluble fraction containing the collagen was freeze dried.

The analyses were performed on duplicate 0.8mg aliquots of the purified collagen using a Sercon continuous flow 20–22 Isotope Ratio Mass Spectrometer. Results are quoted as δ values vs. PDB and AIR for C and N respectively. Duplicate collagen analyses showed typical reproducibility of 0.05‰ for δ^15^N and 0.20‰ for δ^13^C. The CN molar ratio was monitored as a control for contamination and fell within the acceptable limits of 2.9–3.6.

### Radiocarbon dating

The individuals from Armoy [10,26] and Ardreigh [13] had both been previously dated so it was only necessary to obtain radiocarbon dates from the Golden Lane skeletons. Samples of rib were submitted to the ^14^CHRONO Centre of the School of Natural and Built Environment of Queen’s University Belfast, for radiocarbon dating.

## Results

### Osteology

Summary details of the four previously published cases are provided in Table 2.

**Table 2:** Summary of the osteological characteristics of the previously published individuals from Golden Lane and Armoy.

Site	Context	Completeness	Age (years)	Sex	Pathology Cranial	Feet and lower legs
Armoy	Sk171	< 25% (only feet)	Adult	?	-	Diaphyseal remodelling
						Knife edge deformity
						Cup and peg deformity
						Dorsal tarsal exostoses
						Inflammatory pitting
Golden Lane	SkCXLVIII	75–100%	13–18	Pr male	None	Diaphyseal remodelling
						Destruction of extremities MT and P
						Inflammatory pitting
						Reactive new bone formation
Golden Lane	SkCXCV	75–100%	35–50	Male	Rhino-maxillary syndrome	Diaphyseal remodelling
						Destruction of extremities MT and P
						Knife edge deformity
						Cup and peg deformity
						Dorsal tarsal exostosis
						Reactive new bone formation
Golden Lane	SkCCXXX	75–100%	35–50	Male	Rhino-maxillary syndrome	Minor destruction of extremities MT and P
					Chronic maxillary sinusitis	Reactive new bone formation
					Large lytic lesion on frontal bone	

Pr = Probable, MT = metatarsal, P = phalanges.

**Fig 2:**
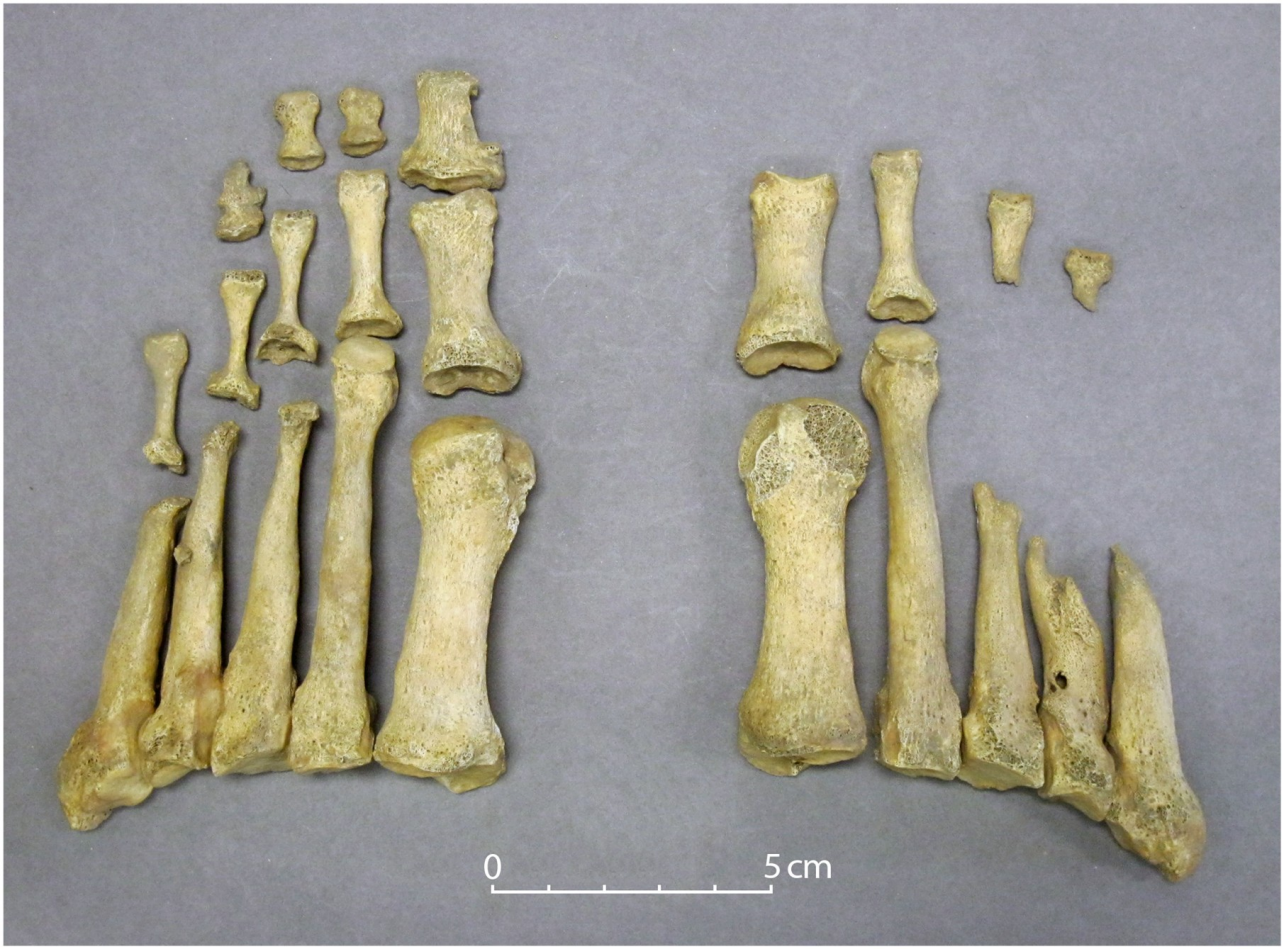
Lesions characteristic of leprosy in the feet of Sk1494 from Ardreigh, Co. Kildare.

The individual from Ardreigh (Sk1494) was a possible male adult of 18–35 years. The post-cranial skeleton was largely complete but the skull was not preserved. Lesions suggestive of leprosy were evident in the bones of the lower legs and feet (Fig 2). In the left foot, diaphyseal remodeling was evident in the third, fourth and fifth metatarsals and a nodule of bone was attached to the anterior surface of the shaft of the left fourth metatarsal. The heads of these metatarsals had been destroyed and corresponding lesions in the third and fourth proximal phalanges had resulted in the “cup-and-peg” deformity. The proximal end of the fifth proximal phalanx also displayed destruction, while possible minor lytic activity was visible at the distal end of the first distal phalanx. Diaphyseal remodeling was evident in the third, fourth and fifth proximal phalanges. Turning to the right foot again, the bones of the first and second digits were largely spared, while the remaining metatarsals had undergone major destruction of their distal halves. Diaphyseal remodeling appears to have resulted in a fracture of the shaft of the fourth metatarsal; the distal end then slipped and became attached to the proximal part of the bone. Three abscess sinuses were evident. The distal ends of the third and fourth proximal phalanges were present, with the proximal aspects having been destroyed as a result of the disease processes. Dorsal tarsal exostoses were notable on the majority of tarsals present, with the naviculars and cuboids most severely affected. Bilateral and pronounced reactive new bone formation, with a spiculed appearance and in the process of healing, was evident on all surfaces of the distal halves of the tibiae and fibulae. Fractures were visible in the neural arches of the eleventh thoracic (unhealed) and fifth lumbar (healed) vertebrae, while a single right rib displayed a healed fracture. It is possible that the disintegration of the architecture of the foot had made the individual more susceptible to accidental injuries.

### Biomolecular study (UoS)

#### *M*. *leprae* screening methods

**Fig 3:**
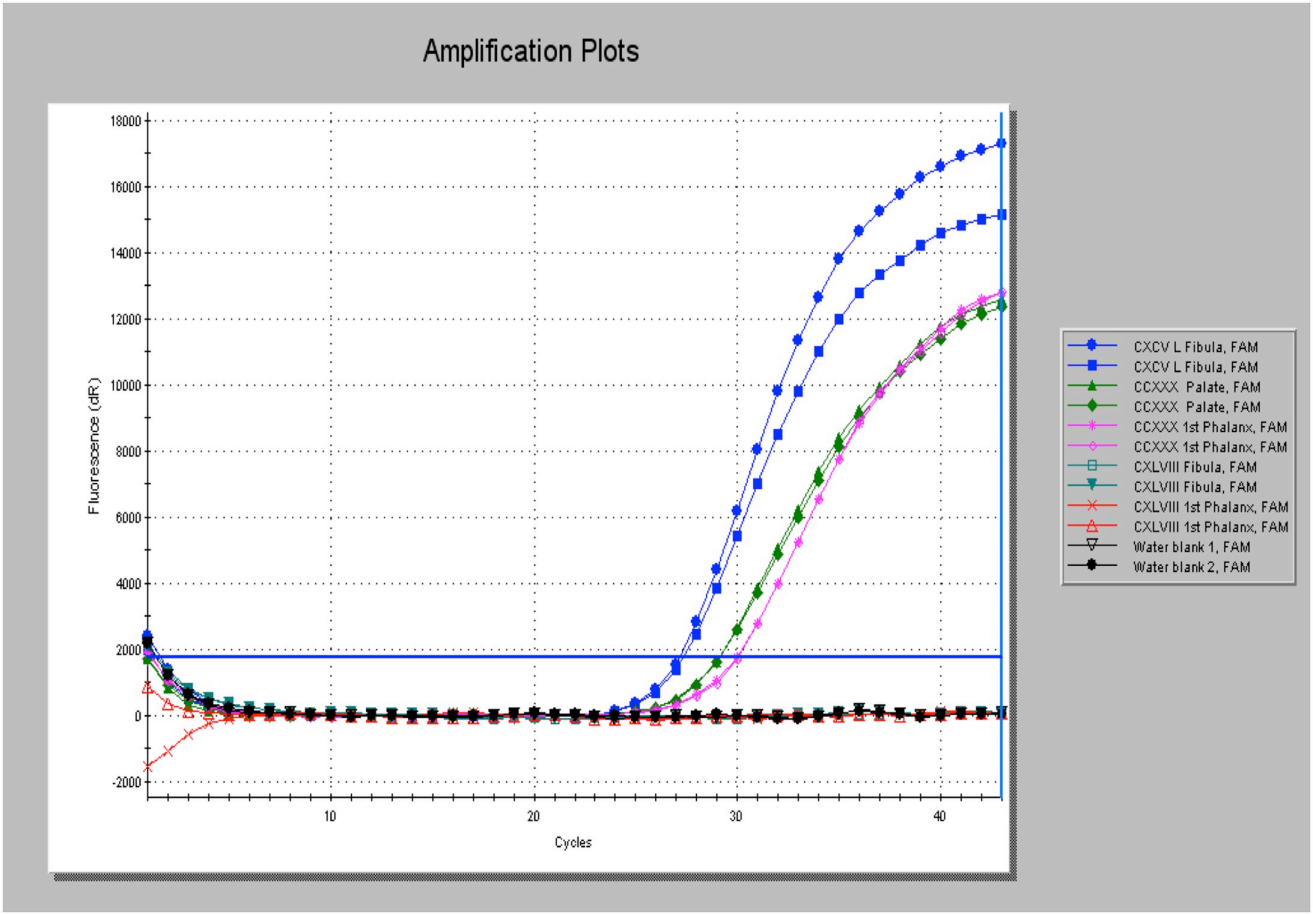
Amplification plots of the multi-copy PCR target RLEP reported with a specific FAM-labelled fluorogenic probe. Products were generated from extracts prepared from SkCXCV (left fibula, blue traces) and SkCCXXX from two extracts (palate, green traces) and first phalanx (fuchsia pink trace). SkCXLVIII was negative using this PCR method, as were the two template blanks run in parallel (black traces).

**Fig 4:**
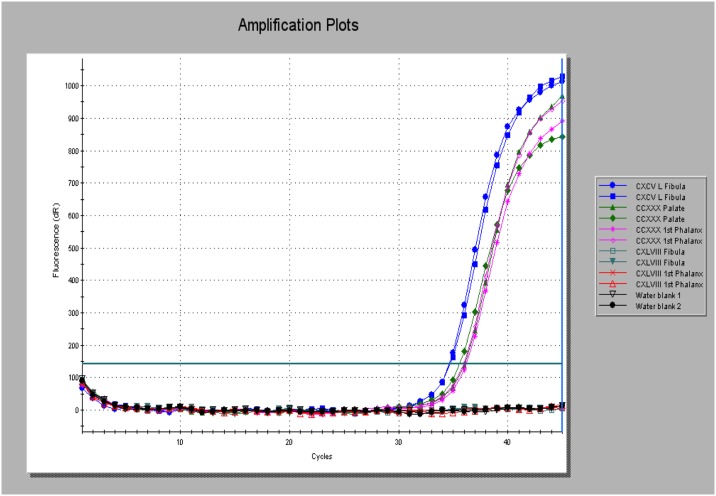
Amplification plots of the single-copy 18kDa gene PCR product reported with a specific JOE-labelled fluorogenic probe. Products were generated from extracts prepared from SkCXCV (left fibula, blue traces) and SkCCXXX from two extracts (palate, green traces) and first phalanx (fuchsia pink trace). SkCXLVIII was negative using this PCR method, as were the two template blanks run in parallel (black traces).

Screening of the Golden Lane cases revealed two robust positives for RLEP PCR reported with EVAGreen^™^ –SkCXCV and SkCCXXX. Both extracts prepared from the latter case was positive, while SkCXLVIII was only weakly positive in one of the two extracts tested, that derived from the fibula (Table 3). Burials SkCXCV and SkCCXXX were also positive for RLEP using the specific FAM labeled reporter probe and also for the single-copy 18-kDa locus (See Figs 3 and 4 and Table 3). No evidence for *M*. *leprae* DNA was found in the cases tested from Armoy and Ardreigh. There was a notable difference between the first and second rounds of sampling in terms of pathogen DNA recovery found in the Golden Lane cases, with the initial pilot study providing more robust evidence and lower Cq values on the PCR real-time platform.

**Table 3:** Summary of screening methods for *M*. *leprae* applied to Irish cases.

Site	Context	Element	Sample (mg)	RLEP PCR EVA Green^™^	RLEP PCR FAM Probe	18-kDa PCR JOE Probe
Golden Lane	SkCCL	Rib	80	-	-	ND
	SkCXLVIII	Distal fibula	80	±	-	-
	SkCXLVIII	R proximal foot phalanx	80	-	-	-
	SkCXCV	L distal fibula	80	+	+	+
	SkCCXXX	Palate (R+L)	80	+	+	+
	SkCCXXX	L proximal first foot phalanx	80	+	+	+
Ardreigh	Sk1494 (1)	R proximal foot phalanx	80	-	ND	ND
	Sk1494 (2)	R fibula	80	-	ND	ND
Armoy	Sk 171	Tarsal	30	-	-	ND

+ = PCR positive, ± = weak positive,— = PCR negative, ND = not determined.

#### SNP genotyping of positive cases

A series of phylogenetically informative SNP PCRs were applied to Golden Lane cases SkCXCV and SkCCXXX. A summary of the sequencing data obtained from these is shown in Table 4. All amplicons generated were of expected sizes. The data revealed that SkCXCV was a type 3I-1 strain and that SkCCXXX belonged to the rarer, type 2F lineage [21]. No novel *M*. *leprae* polymorphisms were detected and all sequences exactly matched those previously reported in public databases.

**Table 4:** Summary of SNP genotyping from Golden Lane positive cases.

Locus: TN positio^1^	Amplicon Size (bp)	SkCXCV	Inference	SkCCXXX	Inference
SNP 1: 14,676	121	C		C	
SNP 2: 1,642,875	122	T		T	
SNP 3: 2,935,685	117	C	Main type 3	A	Main type 2
SNP 4: 413,903	120	G	Subtypes I-K	G	Subtypes E-H
SNP 5: 591,857	107	C	Subtypes I-L	C	Subypes E-H
SNP 6: 1,133,492	121	T	Subtype I	T	Subtypes E-H
SNP 7: 2,312,066	101	C	Subtypes I or J	C	Subtypes E-H
SNP 8: 7,614	109	T	Subtype I	C	Main types 1–4
SNP 9: 1,113,923	117	G	Main type 3	A	Main types 1,2 or 4
SNP 10:1,104,235	117	ND		C	Subtypes E or F
SNP 11: 3,102,787	139	ND		C	Subtypes F-H
Indel 17915	120	1 copy	3I	2 copies	Not 3I
11 bp repeat					
SNP 12: 1,527,056	101	G	3I-1	G	
**Genotyping**			**3I-1**		**2F**

^1.^ SNP numbering denotes position in the amended TN genome [19].

#### VNTR typing

We undertook multiple loci variable number tandem repeat (VNTR) analysis of three loci [44] (Table 5). The two cases were identical at two of the three loci but differed at the (GTA)9 locus, with SkCXCV having an extra copy of this triplet repeat. We have not previously encountered *M*.*leprae* strains with this combination of SNP and VNTR polymorphisms.

**Table 5:** VNTR analysis at the three chosen polymorphic loci.

Individual	TTC (AGA)20	(GTA)9	ML0058 (21–3)
SkCXCV	10	10	2
SkCCXXX	10	9	2

#### Screening for *M*. *lepromatosis* DNA

No evidence for this pathogen was found in any of the Irish cases with osteological signs of leprosy or in the individual used as a control in the genotyping experiments, SkCCL. In separate experiments (not shown) it was found that the primers amplified the expected target of 135 bp from *M*. *lepromatosis* DNA partially purified from an infected red squirrel (*Sciurus vulgaris*). This DNA was kindly supplied by Dr. Charlotte Avanzi, of The Global Health Institute, École Polytechnique Fédérale de Lausanne, 1015 Lausanne, Switzerland.

#### Screening for *M*. *tuberculosis* complex DNA

All five cases tested negative for MTB complex DNA using the IS*1081* multi-copy marker.

#### Evidence for Human DNA preservation

Four of the six Irish skeletons included in the study provided evidence for human DNA survival, with only SkCCXXX and the case from Armoy (Sk171) being negative for both methods (Table 6). Preservation was poorer in cases SkCCL and SkCXLVIII, which showed a greater degree of fragmentation, as judged by the failure of the PCR for the larger mtDNA template of 279 bp. Attempts to confirm or determine the sex of the individuals using the amelogenin PCR procedures were unsuccessful, and this is very likely due to the poor survival of single copy nuclear DNA compared to the multi-copy nature of mtDNA.

**Table 6:** Screening for human DNA survival (UoS).

Site	Context	mtDNA	mtDNA	Amelogenin	Amelogenin
		116 bp	289 bp	105/290bp	106/112bp
Golden Lane	SkCCL	+	-	-	-
	SkCXLVIII	+	±	-	-
	SkCXCV	+	+	-	-
	SkCCXXX	-	-	-	-
Ardreigh	Sk1494	++	+	-	-
Armoy	Sk171	-	-	-	-

#### Second Centre aDNA confirmation

A series of PCRs were undertaken at the University of East Anglia to confirm the key aDNA observations made at University of Surrey (Table 7). Confirmation at a second centre is a key criterion for authentication and publication. The laboratory at UEA confirmed the presence of *M*. *leprae* DNA in the two Golden Lane cases with positive determinations at the University of Surrey. The RLEP amplicon was sequenced at UEA to confirm product identity. The UEA laboratory was also able to confirm the UoS mtDNA results for SkCCXXX and SkCXCV. Additionally, UoS detected mtDNA in the WGS control burial SkCCL and SkCXLVIII.

**Table 7:** Comparison of key biomolecular findings at UoS and UEA laboratories.

Individual	UoS RLEP (Probe)	UEA RLEP	UoS mtDNA 116 bp	UEA mtDNA 116 bp
SkCCL	-	-	+	-
SkCXLVIII	-	-	+	-
SkCXCV	+	±	+	+
SkCCXXX	+	+*	-	-

* = confirmed by sequencing.

#### Whole genome sequencing (UoS)

Whole genome sequencing was performed at Genewiz with their HiSeq^™^ X Ten platform. The reads were quality controlled and aligned to the *M leprae* TN genome using Bowtie2 [54]. Duplicate reads were removed with Mark- Duplicate script and SNPs assigned. The criteria for inclusion in the SNP/indel list was that polymorphs had to be outside of annotated repeat regions, to have a read depth of more than three reads in the sample but less than three reads in the control sample SkCCL, to have a QUAL score of > = 30 (not required for longer indels) and appear in more than 75% of reads. Indels had an additional requirement that all qualifying reads had to span the indel.

Sequence data was obtained for all three individuals (Table 8). Read lengths were consistent with the data being derived from ancient DNA. The two leprous individuals, SkCXCV and SKCCXXX, had higher percentages of read aligning to the *M*. *leprae* genome than the control, SkCCL, and lower rates of alignment to human genomes consistent with the results from the PCR typing. Unfortunately, due to the relatively small number of reads recovered from SkCXCV and SKCCXXX, coverage in the reconstructed genomes remained low and sporadic and so confirmation of the SNP, Indel and VNTR typing was not possible.

**Table 8:** Sequence data obtained for the whole genome sequence study.

Individual	Total number of reads	Percentage of reads aligning to the *M*. *leprae* TN genome	Percentage of reads aligning to the human genome	Percentage Genome coverage *	Mean length of aligned reads ± SD
SkCCL	37,038,101	0.3	48.9	1.1	89.3±13.3
SkCXCV	6,583,167	6.8	9.9	1.0	91.6**±**13.2
SkCCXXX	193,108	9.3	5.1	0.52	91.5**±**12.1

*Genome coverage requires sites to have > = 3 reads, > = 75% concurrence, and an MQ score > = 30. SD—Standard Deviation.

#### Isotopic analyses

The results of the strontium isotopic analyses are included in the S3 File. The results of the carbon and nitrogen isotopic analyses are presented in Table 9, while Table 10 includes the results of the oxygen isotope analyses.

**Table 9:** Results of the carbon and nitrogen isotopic analyses on ribs and long bones.

Site	Individual	Ribs			Long bone cortex		
		d^15^N_AIR_ ‰	d^13^C_PDB_ ‰	C:N	d^15^N_AIR_ ‰	d^13^C_PDB_ ‰	C:N
Golden Lane	SkCXLVIII	12.09	-19.21	3.27	11.68	-19.12	3.23
	SkCXCV	12.86	-19.9	3.27	9.68	-20.73	3.26
	SkCCXXX	11.12	-19.56	3.22	10.82	-19.79	3.23
Ardreigh	Sk1494	12.67	-20.22	3.22	-	-	-

The long bone fragment of Sk1494 from Ardreigh was not run due to poor preservation.

**Table 10:** Carbon and oxygen isotopes of structural carbonate (18Oc) in the tooth enamel. For comparison this has been converted to phosphate equivalent values (18Op) using Chenery *et al* (2012) [52] from which drinking water (dw) equivalent values have been calculated according to Longinelli (1984) [55] and Daux *et al* (2008) [5,1].

Individual	d^13^C_c VPDB_ ‰	d^18^O_c VPDB_ ‰	d^18^O_c VSMOW_ ‰	d^18^O_p VSMOW_ ‰	d^18^O_dw long_	d^18^O_dw daux_
SkCXLVIII	-14.11	-5.49	25.26	16.39	-9.23	-8.9
SkCXCV	-14.03	-3.91	26.89	18.07	-6.65	-5.99
SkCCXXX	-13.59	-3.64	27.16	18.35	-6.22	-5.5

NB—Tooth enamel was not available for Sk1494 from Ardreigh.

### Radiocarbon dating

The carbon: nitrogen values for the Golden Lane samples (Table 11) all fell within the recommended values for atomic values reported at the 14CHRONO Centre (2.9–3.5). The radiocarbon dates for all sites included in the study are included in Table 11 and presented in the S4 File.

**Table 11:** Radiocarbon dates derived from the individuals included in the study.

Lab code	Site	Individual	C:N ratio	^14^C Age BP	Cal AD (probability)	
					1 sigma (68.3)	2 sigma (95.4)
UBA-30811	Golden Lane	SkCXLVIII	3.15	1052 ± 36	970–1022 (0.7)	939–1030 (0.8)
UBA-30812	Golden Lane	SkCXCV	3.19	851 ± 30	1163–1221 (0.7)	1152–1260 (0.9)
UBA-30810	Golden Lane	SkCCXXX	3.16	888 ± 33	1151–1208 (0.5)	1039–1219 (1.0)
					1050–1083 (0.2)	
					1126–1136 (0.1)	
SUERC-39778	Ardreigh	Sk1494	3.1	685 ± 35	1275–1303 (0.5)	1264–1319 (0.6)
					1366–1383 (0.3)	
GRA-11985	Armoy	Sk171	-	370 ± 50	1453–1522 (0.4)	1445–1637 (1.0)
					1575–1625 (0.3)	

## Discussion

We examined five human skeletons derived from inhumation burials from Ireland, with lesions indicative of leprosy. Two of these provided strong biomolecular evidence for the causative pathogen, *Mycobacterium leprae*. A third skeleton, SkCXLVIII, the remains of an adolescent individual from the same site at Golden Lane, Dublin, was weakly positive in one of two extracts prepared from a sample taken from a fibula. Due to the poor DNA preservation in this skeleton, however, it was not possible to take this observation further.

In contrast, the preservation of DNA in the other two Golden Lane individuals, SkCXCV and SkCCXXX, was sufficient to allow detailed genotyping of the strains by conventional SNP and VNTR analysis of informative loci. Interestingly, these two cases were found to belong on separate branches of the *M*. *leprae* phylogenetic tree. The radiocarbon dates derived from these two individuals are suggestive that they were broadly contemporary, thereby suggesting that the two strains co-existed in Dublin during the 12^th^ to mid-13^th^ centuries.

Burial SkCXCV was a type 3I-1 strain, a lineage found in several ancient cases which have been excavated from archaeological sites in Britain [20,21] and Europe [Denmark and Sweden] [56]. A Scandinavian source for this lineage is therefore one possibility.

The earliest genotyped case in Britain, dating to the early 5^th^ century from Great Chesterford, Essex [6], is also an early type 3I. The strain persisted in Britain from the Anglo-Saxon period right up to the 16^th^ century [21] and was likely carried to the New World by early colonists. Strains descended from this lineage (3I-2-v1) are still present in the southern United States, in states such as Louisiana and Texas, in both infected humans and armadillos [57].

**Fig 5:**
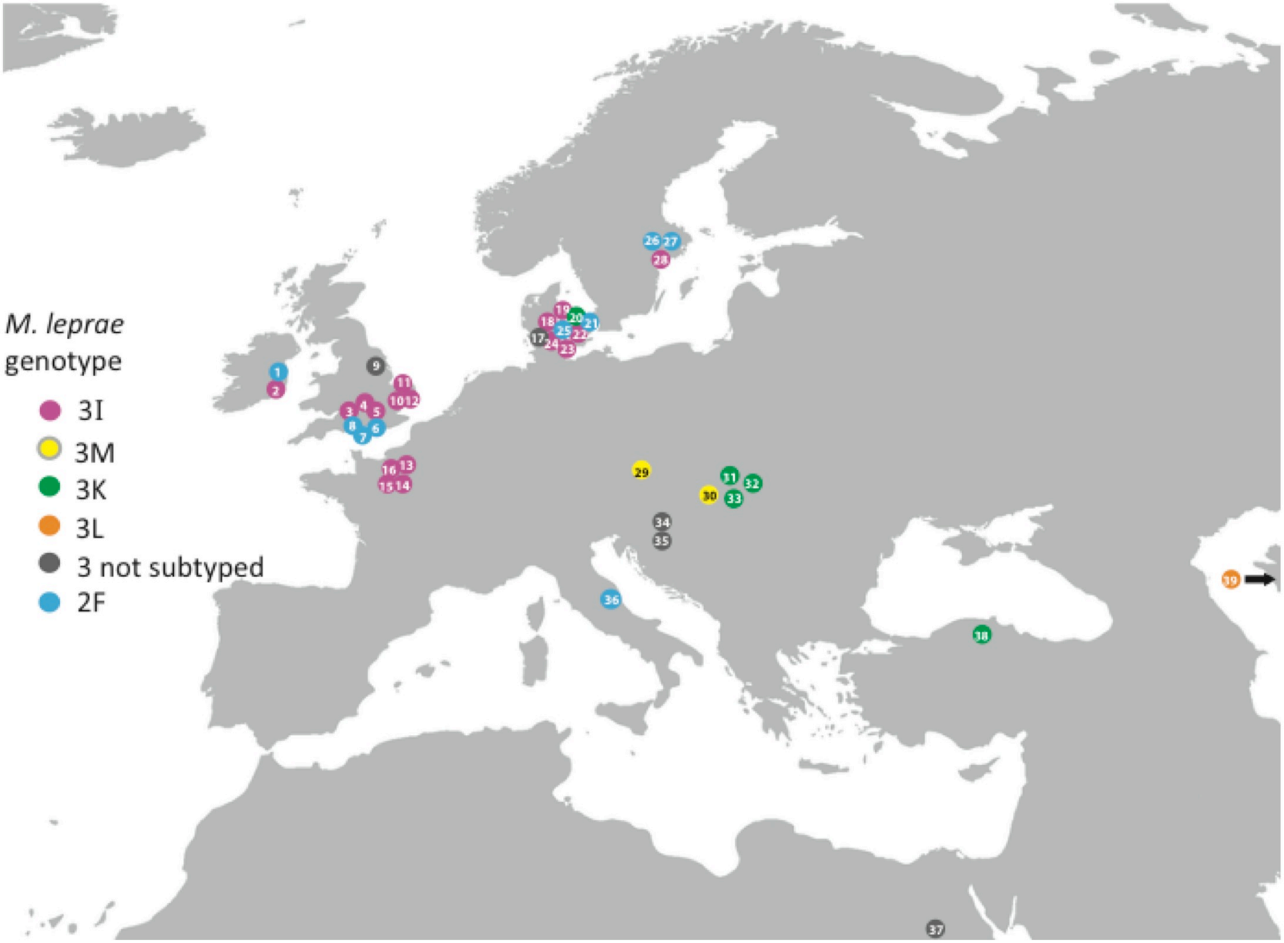
Map summarizing data derived from approximately 40 archaeological cases studied by conventional genotyping or whole genome sequencing (S5 File). The Figure was modified from a blank map of Europe obtained from: https://upload.wikimedia.org/wikipedia/commons/3/37/BlankMap-Europe-v3.png Attribution: ShareAlike 3.0 Unported (CC BY-SA 3.0). Author:User:Roke-Commonswiki.

The similarity in distribution of leprosy genotypes between Britain and northwestern Europe, compared to those known in central Europe, is evident in the map plot shown in Fig 5. This summarizes data from approximately 40 archaeological cases studied by conventional genotyping or whole genome sequencing and includes data derived from 10 cases recently examined by Schuenneman and colleagues [22]. These latest additions confirm the general preponderance of 3I and 2F genotypes in northwestern Europe, but also indicate occasional additional genotypes. For example, a type 3K (or Branch 0) from the St Jørgen cemetery in Odense, Denmark, and a type 2F from the 6^th^-8^th^-century Vicenne-Campochiaro necropolis in Molise, central Italy.

Since the present study of Irish leprosy was begun, the 3I lineage of *M*. *leprae* has also been found in the Brownsea Island, England, population of red squirrels (*Sciurus vulgaris*) [24]. This intriguing observation shows that the pathogen can now cause disease in a third species, opening up the possibility of a zoonotic spread from squirrels to humans (or vice-versa) in past and present populations. It is possible to envisage this happening in the days when red squirrel skins were an important export from Ireland in the heyday of the fur trade [58].

The second strain to be found, in SkCCXXX from Golden Lane, was a type 2F isolate. Previously, type 2s have been identified as a likely precursor strain that migrated from the Middle East to India and South-East Asia, subsequently evolving to type 1 strains. Present-day locations of this genotype include Turkey and Iran. This lineage has also been identified in two individuals, Sk8 (early 11^th^-12^th^ century) and Sk14 (mid-10^th^-11^th^ century), from the *leprosarium* of St Mary Magdalene, Winchester. Economou and colleagues [56] also reported lineage 2 strains in medieval Scandinavia. The presence of a second lineage at the Golden Lane cemetery may indicate a separate incursion of leprosy, probably originally from a different geographical origin. Whilst 2F subtypes of *M*. *leprae* are relatively rarely found amongst extant strains, they are emerging as one of two more commonly encountered isolates in archaeological material, although admittedly, the numbers studied by aDNA analyses remain relatively small. As already mentioned, a type 2F was amongst isolates recently described [22] from body T18, from the Vicenne-Campochiaro necropolis in Molise, central Italy, thereby extending the known range of this genotype to 6^th^–8^th^-century Europe.

Radiocarbon dating from the present study (and the references cited above) demonstrates that both strains were contemporaneous, but information on how long each persisted into the later Medieval period is still lacking.

The other two osteological cases of leprosy, those from Ardreigh and Armoy, were both negative for *M*. *leprae* DNA. They were also negative for the second possible pathogenic agent of leprosy, *M*. *lepromatosis*. In the case of the Armoy remains, this could be due to a lack of any DNA survival as no human mtDNA could be detected either. Mitochondrial DNA was present in the Ardreigh skeleton, however, thereby indicating that DNA from either pathogen should have been found if it had been present. It has often been observed than mycobacterial DNA persists even when human DNA has degraded, and this was seen in the current project with SkCCXXX. It is possible that the disease had run its course in the individual and that the leprosy bacteria were no longer present in the body of the individual, or at least in the bones sampled. Alternatively, the individual may not have suffered from the disease. The lesions are osteologically convincing for leprosy but other conditions, including sarcoidosis and advanced rheumatoid arthritis, can result in similar lesions in the hands and feet. The paucity of primary osteoarthritic lesions in the remains of the individual make rheumatoid arthritis unlikely [59]. In sarcoidosis one would also expect cyst-like lesions and a honeycomb pattern at the ends of the phalanges in addition to other lesions throughout the skeleton [60], none of which was evident in the Ardreigh individual. Another potential differential diagnosis is diabetes mellitus in which neuropathic osteoarthropathy of the feet is very common but typically involves the medial side earlier and more frequently than the lateral aspect [61]. This is not the pattern in the Ardreigh individual in which only the lateral aspects of the feet were affected. It is unfortunate that the cranium of the individual had not been preserved since this might have helped resolve the situation.

**Fig 6:**
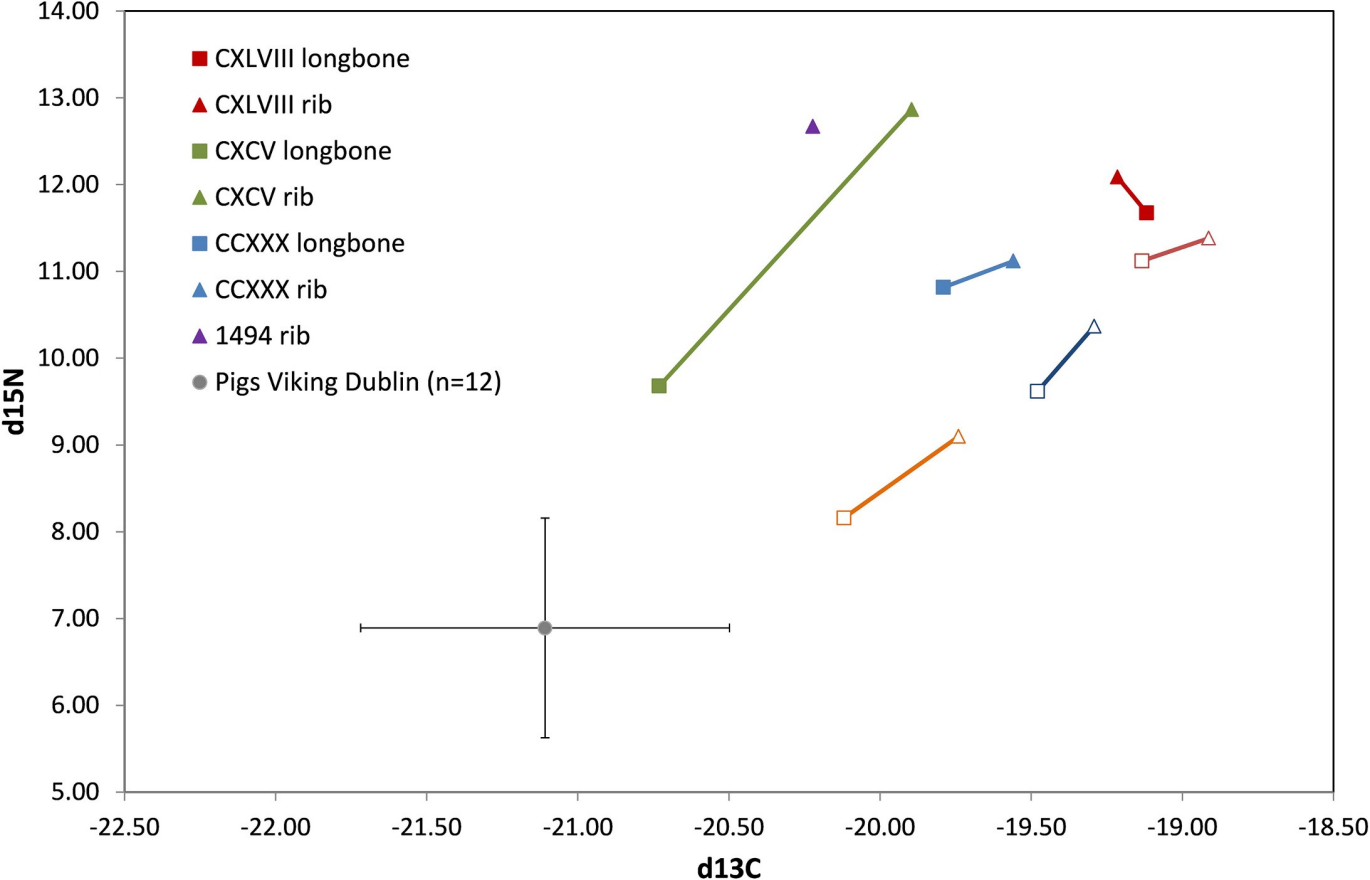
Carbon and nitrogen isotopic data plotted as rib-long bone pairs (closed symbols). Additional data from the leprosy hospital of St Mary Magdalene, Chichester, England, is included for comparative purposes (open symbols).

The sampling of ribs and long bones (femora and humeri) allows an assessment of diet from different times in an individual’s life. Collagen in long bones represent the diet integrated over the last 10–20 years of life, and the ribs around five years. Fig 6 shows the carbon and nitrogen isotopic data plotted as rib-long bone pairs, with additional data from the leprosy hospital of St Mary Magdalene, Chichester, England, for comparison.

The pattern is broadly similar for the two datasets with the ribs more enriched in carbon and nitrogen compared to the long bones. An enrichment of carbon and nitrogen isotopes is usually associated with an increased consumption of marine resources or, for moderate enrichment (δ^13^C<1‰, δ^15^N<4‰), access to greater amounts of terrestrial meat protein. A comparison with published values for pigs from Viking Dublin [62] shows a maximum isotopic enrichment of the ribs over the mean of the pigs of +6‰ in nitrogen and nearly +2‰ in carbon, which is too great to represent a trophic level effect due to the consumption of terrestrial animals. Furthermore, pigs are likely to have eaten some domestic waste and are therefore probably enriched compared to terrestrial herbivores such as cattle and sheep. As such, it seems probable the diet involved the consumption of a component of marine protein. The isotopic enrichment in the ribs indicates that it has occurred later in life, perhaps as the result of care for leprosy sufferers, and the increased marine consumption may perhaps be due to religious traditions of substituting fish for meat on certain days.

The three individuals have different strontium isotope values in the teeth, with means (integrated along the profile) of 0.7092, 0.7140 and 0.7112 for SkCXLVIII, SkCXCV and SkCCXXX respectively. The ‘local’ range for Dublin can be estimated from published values of humans and fauna. Knudson *et al*. (62) found the radiogenic strontium isotope values of 22 Viking individuals in Dublin (AD 800–1200) to range from 0.70824 to 0.71099, with a mean of 0.70975 ± 0.00139 (2σ). While this may also include immigrants, the value was similar to the mean of 11 (presumably less mobile) pigs with ^87^Sr/^86^Sr = 0.70943 ± 0.00144. Using these values, and published values for other fauna, Knudson *et al*. [62] estimate the strontium isotopic ‘local range’ for Dublin to be 0.7084–0.7106. The strontium value of SkCXLVIII falls within this range and is consistent with a local origin for this individual. The other two individuals have more radiogenic strontium values, however, but it is difficult to pinpoint the precise origin of an individual because the geologies that define the strontium isotope ratio are rarely unique to a single region.

Individual SkCCXXX has strontium isotope values that match bioavailable values for parts of Northern Ireland, Eastern Wales and the British Midlands (see Figs A3-C3 in S3 File), so need not have an origin at a great distance from Dublin. The strontium value of SkCXCV, however, is unusually radiogenic (0.7140). While ^87^Sr/^88^Sr values of up to 0.7222 are reported for small areas in the survey of the strontium biosphere of Britain undertaken by Evans and colleagues [63], values of c. 0.7140 are not reported; this is also the case for a recent survey of strontium values in Ireland [62]. Highly radiogenic values (>0.713) are also rare in continental Europe, but are reported in the Rhine Massif, the ore mountains of southern Poland and the Carpathians, Norway and Sweden [64,65]. While we cannot rule out a central European origin, the links between Medieval Dublin and the Norse world would make it reasonable, on the basis of the strontium isotopes, that individual SkCXCV is of Scandinavian origin.

The combination of oxygen with strontium isotopes is often used to narrow down possible origins. The ‘map’ of oxygen isotopes in modern spring and well waters (see Fig D3 in S3 File) shows values in Ireland of between -4 and -8‰, which is consistent with the measured values for SkCXCV and SkCCXXX. SkCXLVIII which had strontium isotope values consistent with a local origin, however, has drinking water equivalent values more depleted (c. -9‰) than those found in Ireland which suggest an origin further east. Areas with both strontium in the range 0.7090–0.7095 and oxygen of around -9‰ are found in the vale of York, central Europe, Denmark and southern Sweden, again raising the possibility that this individual may be of Scandinavian origin. As discussed above, the radiogenic strontium values for SkCXCV, are also consistent with a Scandinavian origin (and certainly exclude an Irish origin), although the oxygen isotope value for this individual is less clear cut. Typically, drinking water equivalent values for southern Scandinavia would be -7 to -10‰. According to Lécolle’s survey [66], however, coastal southern Norway has oxygen values less than -7‰ which makes this a possible location of origin for SkCXCV.

Radiocarbon dating of the Golden Lane individuals revealed that SkCXLVIII is of early 10^th^/early 11^th^ century date and predated the other two individuals with the disease from the burial ground, one of whom dated to the early 11^th^ to early 13^th^ centuries (SkCCXXX), while SkCXCV was of mid-12^th^ to mid-13^th^ century in date. As such, SkCXLVIII would have post-dated the arrival of the Vikings in Dublin in the AD 840s and would have lived in a Hiberno-Norse world. St Michael’s church (later to have the le Pole suffix added in reference to its proximity to ‘the pool’) was founded around AD 1000 and is thought to have started as a proprietary church [25]. The interments at the site generally followed standard Christian burial practices, with the body extended and supine with the head to the west. This was the case for both SkCXCV and SkCCXXX and is suggestive that those who buried the individuals followed Irish cultural norms [11]. This is particularly interesting for SkCXCV, for whom the isotopes suggest a Scandinavian origin, and may be the first definitive identification of a Hiberno-Norse individual. The burial practice for the earlier SkCXLVIII was atypical and the adolescent was buried in a semi-flexed position with the head to the west. It is possible this position was related to the age and/or potential physical impairments suffered by the individual as a result of his disease status [67].

It may have been a chance event that led to the burial of leprosy sufferer SkCXLVIII in the burial ground but the later deposition of two further individuals with the disease seems too much of a coincidence. The earliest reference to the foundation of St Stephen’s leprosarium occurs in the Dublin Corporation Records for AD 1230 [9] although it has been suggested it may have been founded by AD 1192 [25]. This site is located only some 300 m from the Golden Lane site and, given their dates, it is possible that SkCXCV and SkCCXXX would have been alive after it had been founded.

Both the church of St Michael and the hospital of St Stephen lie outside the Medieval town walls in a rural environment near St Kevin’s Gate (see Fig 1). It is tempting to suggest that from potentially as early as the early 10^th^ century the area had some association with leprosy which was later formalised through the establishment of the hospital of St Stephen. The content of the Dublin Corporation Records of AD 1230 in relation to St Stephen record an agreement in the king’s court between the master and inmates of the hospital with Geoffrey and Sara Tyrel related to a land conveyance which then became known as *Baile na Lobhar* or Leperstown. The people of Dublin are considered to have founded the hospital which was then run as a civic institution [9]. The isotopic information is suggestive that SkCXLVIII was of Viking decent, while the later SkCXCV may also have had Scandinavian roots and SkCCXXX was unlikely to have been native to Dublin, perhaps having originated in the north of Ireland or central Britain. It is possible there was a longstanding presence of leprosy in the vicinity of St Michael le Pole that eventually led to the foundation of St Stephen’s hospital, although there is also the possibility these individuals chose to be buried at St Michael le Pole, or indeed were excluded from St Stephen’s, for cultural reasons. The records are suggestive that St Stephen’s was an Anglo-Norman foundation that operated under their regulations [9]. As potential non-locals it may have been the case that they were excluded by the hospital master and inmates or indeed had greater cultural affinity with the older Hiberno-Norse foundation. Alternatively, perhaps an arrangement had been in existence between those managing the two institutions that resulted in the utilization of the St Michael le Pole burial ground and indeed it is perhaps significant that excavations in the vicinity of St Stephen have not yet revealed any osteological evidence for leprosy [68].

Unfortunately, it was not possible to obtain information about the childhood origins of the individuals from Armoy and Ardreigh due to a lack of dentition. The individual from Ardreigh dated to the mid-13^th^ to early 14^th^ century, while that from Armoy was of mid-15^th^ to early 17^th^ century in date. As such, it is difficult to be precise about the geographical source of infection from which these individuals caught the disease since so many scenarios are possible due to the high levels of connectivity of Ireland with the rest of the world during these centuries.

## Conclusions

Three out of five probable cases of Medieval Irish leprosy were found to contain DNA from the pathogen *M*. *leprae*. In two of these it was possible to obtain both main genotype and subtype. The strains were identified as types 2F and 3I, lineages identical to those seen previously in southern Britain and Scandinavia. Isotopic analysis excluded an Irish origin for individual SkCXCV (strain 3I) who lived during the 12^th^–13^th^ centuries and may well have spent his formative years in Scandinavia, possibly in southern Norway. The fact that this individual was interred following the norms for Irish Christian burial is suggestive that he was a genuine Hiberno-Norse individual—a Scandinavian who had adopted Irish cultural practices. Individual SkCCXXX (strain 2F), was radiocarbon dated to the 11^th^–13^th^ centuries and, whilst not local to Dublin may have originated in Northern Ireland, Eastern Wales or the central midlands of Britain. *M*. *Leprae* DNA was also identified in SkCXLVIII, an individual with strontium isotope values consistent with a local origin but drinking water equivalent values that correspond to the vale of York, central Europe, Denmark and southern Sweden, again raising the possibility that this individual may be of Scandinavian origin. The three cases from Golden Lane provide a tantalizing glimpse into the cosmopolitan nature of Dublin in Medieval times—none of the three men appear to have been local to Dublin and, while one may have been British or Irish, one and perhaps two were of Scandinavian origin. The radiocarbon date for SkCXLVIII (early 10^th^–early 11^th^ century) is suggestive that the area to the south-east of the Medieval town may have had longer associations with leprosy than previously thought and that it had Hiberno-Norse roots.

A multidisciplinary approach, combining pathogen aDNA analysis with geochemical stable isotopes on archaeological individuals with skeletal indicators of leprosy is an instructive way for understanding the global spread of leprosy in antiquity. This study has revealed that despite its location on the western extremity of Europe Ireland and, certainly, Dublin was not isolated from the rest of the world during Medieval times. Multiple strands of archaeological evidence indicate it was a vibrant port town throughout this era, a situation that brought the benefits of wealth but also facilitated the spread of infectious diseases. Indeed, the results of this study are suggestive that a previously overlooked aspect of the Viking legacy was the introduction of leprosy to Ireland.

## Supporting information

S1 File Osteoarchaeological characteristics. (DOCX)

S2 File Sampling strategy. (DOCX)

S3 File Stable isotopes. (DOCX)

S4 File Radiocarbon dating. (DOCX)

S5 File Table of details of sites and studies referred to in Fig 5. (DOCX)
