# Supplementary material for: Leprosy at the edge of Europe—Biomolecular, isotopic and osteoarchaeological findings from medieval Ireland
Source: PLoS One. 2018 Dec 26;13(12):e0209495. doi: 10.1371/journal.pone.0209495 (PMC6306209; doi:10.1371/journal.pone.0209495)
Supplement: S1 File — (DOCX) [file pone.0209495.s001.docx]

**Supporting information S1 file – osteoarchaeological characteristics**

**Golden Lane**

The lesions apparent in the cases from Golden Lane (Buckley, 2008) have already been published but the skeletons were re-examined as part of the current project. The skeletons were all in a very good state of preservation with minimal levels of surface damage and fragmentation and 75-100% completeness (McKinley, 2004).

**SkCCL (Control inhumation without signs of leprosy)**

The individual was a probable female with an age-at-death of 18-35 years.

**Sk****CXLVIII**

The skeleton was 75-100% complete and the individual was a probable male with an age-at-death of 13-18 years. Pronounced *cribra orbitalia* and porotic hyperostosis, caused by nutritional deficiencies and perhaps chronic infection, were evident (Walker *et al*, 2009; Mays 2012). Periosteal reactive new bone formation in various stages of healing and indicative of infection and/or inflammation, was visible on the posterior surfaces of the proximal ends of the ulnae. Lesions suggestive of leprosy were evident on the bones of the lower legs and feet (Fig A1), whereas no characteristic signs of the disease were observed in the rhinomaxillary area.


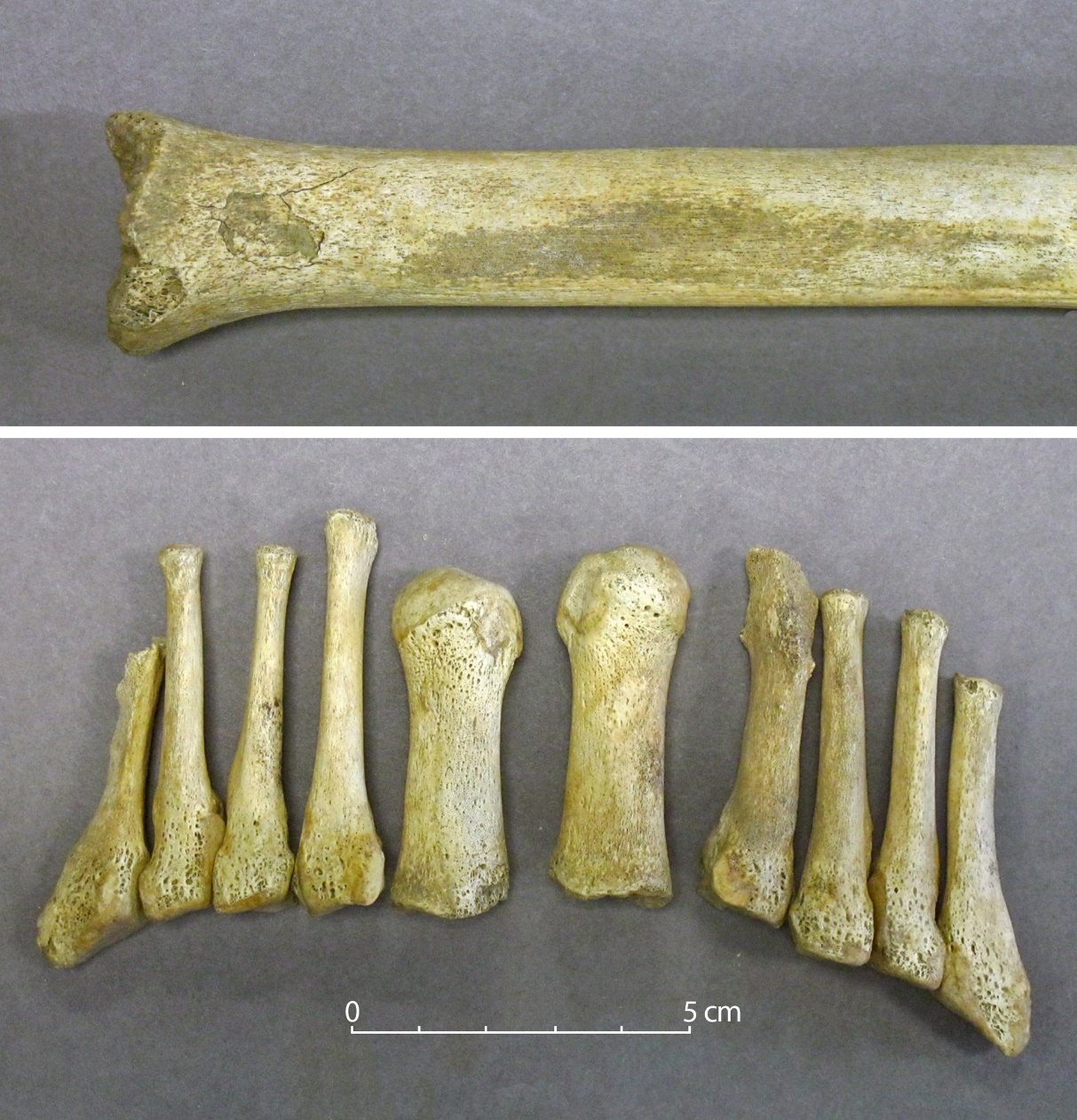


**Figure A1. Osteological lesions related to leprosy in the metatarsals and right tibia of SkCXLVIII from Golden Lane, Dublin.**

The unfused head of the left fifth metatarsal displayed lytic activity and the diaphysis was remodeled, while destruction was also evident at the unfused head of the right second metatarsal and in two proximal phalanges. Periosteal reactive new bone formation was visible on the medial surface of the left first metatarsal. The metatarsals and tarsals generally displayed a pitted appearance which may also be indicative of infection. Periosteal reactive new bone formation, in a variety of stages of healing, was also apparent on the medial and lateral surfaces of the distal halves of the left tibia and fibula and on the medial surface of the distal two-thirds of the right tibia.

**SkCXCV**

The skeleton was 75-100% complete and was that of a male with an age-at-death of 35-50 years. Lesions typical of the rhino-maxillary syndrome of LL were evident and consisted of near-complete destruction of the maxillary alveolar bone adjacent to the incisors, which had all been lost *ante-mortem* (Fig B1). The destruction of the alveolar bone had resulted in the obliteration of the anterior nasal spine and partial perforation of the anterior aspect of the palate, which also displayed a porous surface. The margins of the nasal cavity were notably remodeled.


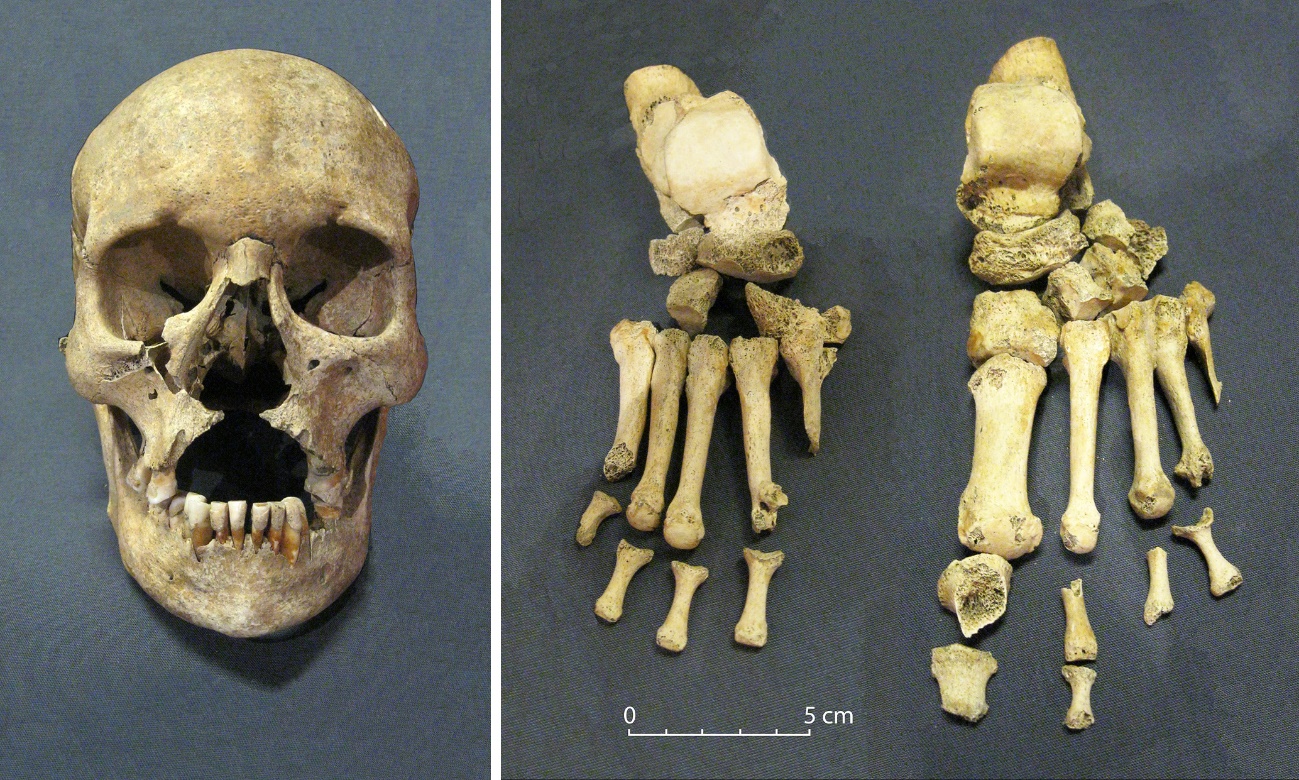


**Figure B1. Osteological lesions related to leprosy in the facial area and feet of SkCXCV** **from Golden Lane, Dublin.**

The head of the left fourth metatarsal displayed lytic activity and corresponding lesions in the proximal phalanx had resulted in the characteristic “cup-and-peg” deformity. Extensive diaphyseal remodeling was evident in the left fifth metatarsal and “knife edge” remodeling, another common lesion in leprosy, was visible at its distal end. A number of the phalanges also displayed some destruction at their distal ends; this was most notable in the first distal phalanx. Dorsal tarsal exostoses were evident on the right navicular. The right foot was less well preserved but its first metatarsal displayed severe resorption of its shaft in addition to the “knife edge” deformity. Destruction of the head was evident in the second metatarsal; a fragment of the head appears to have detached and reattached slightly inferiorly to its normal position. In addition, the metatarsophalangeal joint displayed the “cup-and-peg” deformity. Extensive reactive new bone formation in the process of remodeling was evident on the tibiae and fibulae.

**Sk****CCXXX**

The individual was 75-100% complete and was a male with an age-at-death of 35-50 years. Lesions characteristic of the rhino-maxillary syndrome of lepromatous leprosy were evident and comprised complete resorption of the anterior nasal spine and remodelling of the margins of the nasal cavity (Fig C1). Recession of the maxillary alveolar bone adjacent to the incisors was also apparent but the teeth were still present with the sockets in the mid stages of remodelling. The palate had a notably porous appearance and a possible perforation was evident at its anterior aspect; *post-mortem* damage obscured the full examination of the lytic activity. Evidence of chronic maxillary sinusitis was also evident. A cranial lesion less typical of leprosy comprised a large oval-shaped lesion (40 x 20 mm) in the centre of the frontal bone which had destroyed the outer table of the cranial vault. It had somewhat irregular margins and the interior surface displayed pitting that appeared remodeled and in the process of healing. The endocranial surface of the frontal bone displayed traces of striations that ran antero-posteriorly and appeared to be of long standing and somewhat healed; the cranial vault was also notably thickened (10 mm).


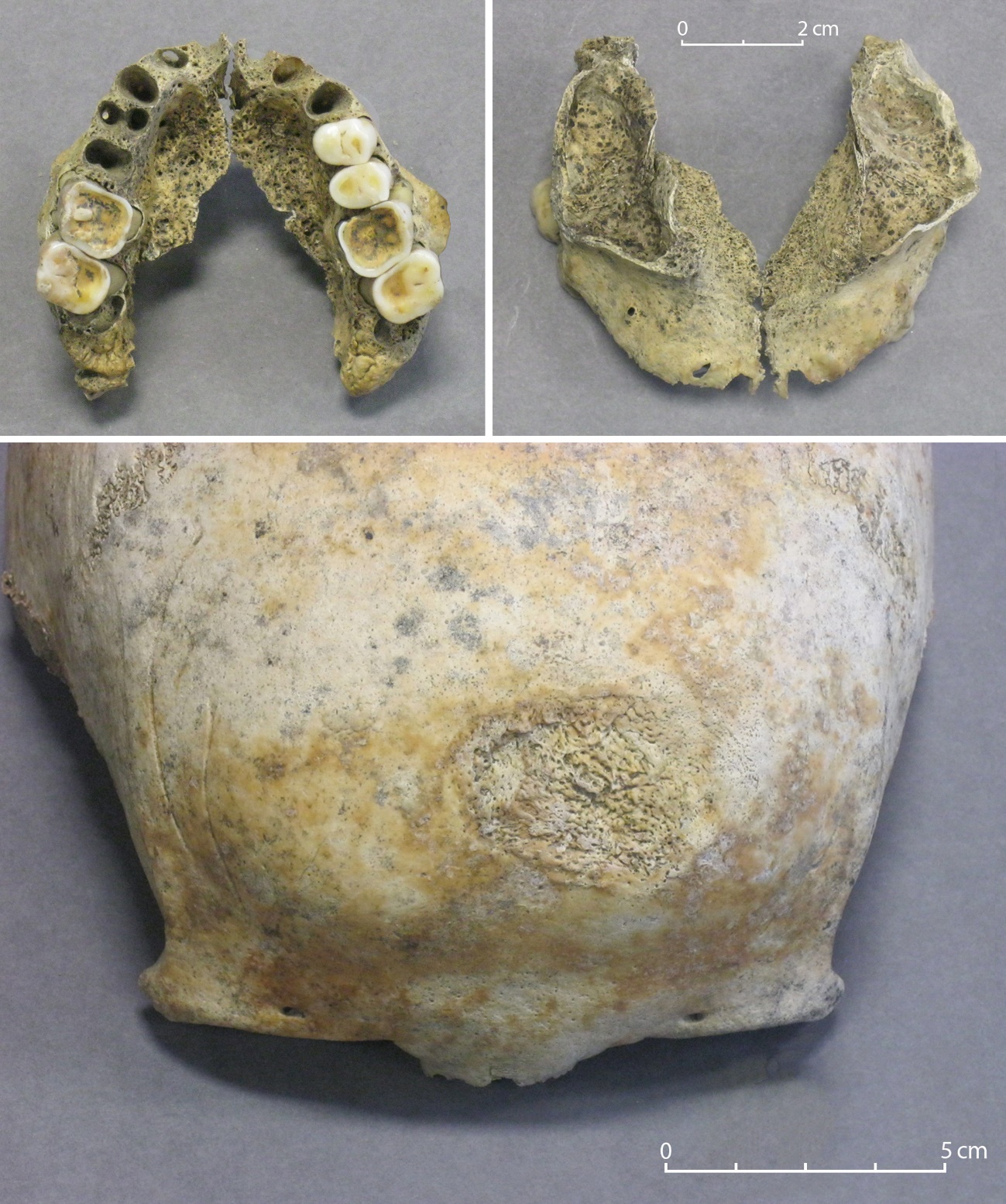


**Figure C1. Osteological lesions related to leprosy in the palate and frontal bone of SkCCXXX** **from Golden Lane, Dublin.**

The feet were relatively unaffected and minor remodeling of the heads of the left second and fourth metatarsals were the only lesions evident. Patches of reactive new bone formation with a healed appearance were evident on the medial and lateral surfaces of the distal halves of the tibiae and fibulae. In addition to displaying marginal osteophytes, the anterior surfaces of the bodies of the fourth and fifth lumbar vertebrae had a pitted, spiculed appearance which may be suggestive of adjacent infectious processes.

**Armoy**

Details of the Armoy case (Sk171) have already been published (Murphy and Manchester 1998; 2002) but the lesions present are detailed below. Only the feet of the individual were preserved but changes indicative of lepromatous leprosy were clearly evident in these (Fig D1).


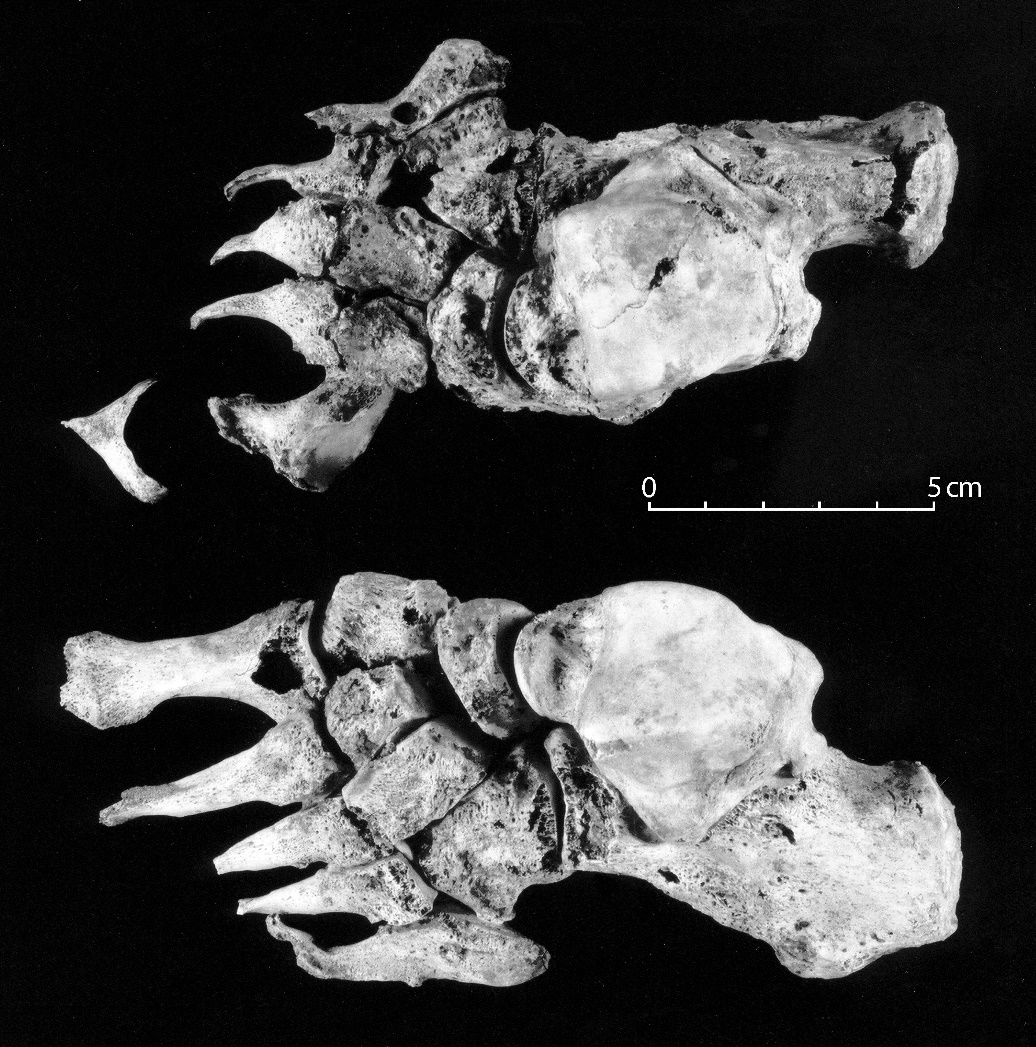


**Figure D1. Osteological lesions related to leprosy in the feet of Sk171 from Armoy, Co. Antrim.**

Diaphyseal remodelling in which the medio-lateral diameter was diminished was apparent in all metatarsals causing them to have a ‘sucked candy’ appearance, while their distal ends displayed the ‘knife edge deformity’. The only phalanx present was the right first proximal phalanx which displayed the ‘cup and peg’ deformity of the first metatarsophalangeal joint. Since the destruction of the first proximal phalanx was so advanced it is highly probable that all of the other phalanges had been destroyed as a consequence of the disease process, rather than not having been recovered during the excavation. Dorsal tarsal exostoses were present on several of the tarsal bones. Surface inflammatory pitting was evident on the dorsal and plantar surfaces of most tarsals and all metatarsals which is indicative of overlying soft tissue infection.

**References**

Buckley L. Outcasts, or care in the community? Archaeol Ire. 2008;22: 26-31.

Mays S. 2012. The relationship between palaeopathology and clinical sciences. In: Grauer AL, editor. A Companion to Palaeopathology. Chichester: John Wiley and Sons, Ltd; 2012. pp. 285-309.

McKinley J. Compiling a skeletal inventory: disarticulated and co-mingled remains. In: Brickley M, McKinley J, editors. Guidelines to the standards for recording human remains (BABAO and Institute of Field Archaeologists Paper No. 7). Reading: BABAO and IFA; 2004. pp. 14-17.

Murphy E, Manchester K. ‘Be Thou Dead to the World’ – palaeopathological evidence for leprosy in Ireland from Armoy, Co. Antrim. Archaeol Ire 1998;12: 12-14.

Murphy EM, Manchester K. Evidence for leprosy in Medieval Ireland. In: Roberts CA, Lewis ME, Manchester K, editors. The past and present of leprosy. Archaeological, historical, palaeopathological and clinical approaches (BAR 1054 International Series). Oxford: Archaeopress; 2002; pp. 193-200.

Walker PL, Bathurst RR, Richman R, Gjerdrum T, Andrushkp VA. The causes of porotic hyperostosis and cribra orbitalia: a reappraisal of the iron-deficiency-anemia hypothesis. Am J Phys Anthropol 2009;139: 109-125.
