# Supplementary material for: Leprosy at the edge of Europe—Biomolecular, isotopic and osteoarchaeological findings from medieval Ireland
Source: PLoS One. 2018 Dec 26;13(12):e0209495. doi: 10.1371/journal.pone.0209495 (PMC6306209; doi:10.1371/journal.pone.0209495)
Supplement: S2 File — (DOCX) [file pone.0209495.s002.docx]

**Supporting information S2 file – sampling strategy**

**aDNA analysis**

The various samples collected for aDNA analysis are detailed in Tables A2 and B2.

**Table A2. First sampling round of burials from Golden Lane, Dublin, and Armoy, Co. Antrim.**

| **Site** | **Context** | **Element** | **1st Sample (mg)** | **Excess (mg)** |
| --- | --- | --- | --- | --- |
| Golden Lane | SkCXLVIII | Distal fibula | 80 | 240 |
|  | SkCXLVIII | R proximal foot phalanx | 80 | 240 |
|  | SkCXCV | L distal fibula | 80 | 180 |
|  | SkCCXXX | Palate (R+L) | 80 | 185 |
|  | SkCCXXX | L proximal first foot phalanx | 80 | 320 |
| Armoy | Sk171 | Tarsal | 30 | - |

In a second round of sampling (Table B2), replicate samples were prepared for whole genome sequencing (WGS), second centre confirmation and final conventional genotyping at UoS. At this time an additional case from Ardreigh, Co. Kildare, Sk1494, was obtained for screening.

**Table B2. Second round of sampling of the Golden Lane, Dublin, cases plus a control from the same site, SkCCL, lacking macroscopic skeletal lesions characteristic of leprosy. Details of the samples analysed for the case from Ardreigh, Co. Kildare, are also included.**

| **Site** | **Context** | **Element** | **1^st^ Sample (mg)** | **Replicated** |
| --- | --- | --- | --- | --- |
| Golden Lane | SkCCL | Rib | 80 | x3 |
|  | SkCXLVIII | R Fibula | 80 | x3 |
|  | SkCXCV | R Fibula (prox) | 80 | x3 |
|  | SkCCXXX | L Radius | 80 | x3 |
| Ardreigh | Sk1494 | R prox foot phalanx | 80 | x3 |
|  | Sk1494 | R Fibula (distal) | 80 | x3 |

**Stable isotope analysis**

**Table C2. Tooth samples removed and analysed for stable isotopes from the Golden Lane, Dublin, and Ardreigh, Co. Kildare, sites.**

| **Site** | **Context** | **Tooth** | **Approx. age of enamel mineralisation (years)^1^** |
| --- | --- | --- | --- |
| Golden Lane | SkCXLVIII | R maxillary M2 | 2.5-8.5 |
|  | SkCXCV | R maxillary M2 | 2.5-8.5 |
|  | SkCCXXX | R maxillary M3 | 8.5-13.5 |
| Ardreigh | Sk1494 | - | - |

^1^The approximate age of the individual for each point in the teeth is estimated using the median crown initiation and crown completion ages given in AlQahtani *et al*, (2010).

**Table D2. Long bone and rib samples removed and analysed for stable isotopes from the Golden Lane, Dublin, and Ardreigh, Co. Kildare, sites.**

| **Site** | **Context** | **Long bone** | **Rib** |
| --- | --- | --- | --- |
| Golden Lane | SkCXLVIII | R midshaft fibula | R midshaft |
|  | SkCXCV | L distal fibula | L midshaft |
|  | SkCCXXX | L radius | R midshaft |
| Ardreigh | Sk1494 | R distal fibula | R midshaft |

**References**

AlQahtani SJ, Hector MP, Liversidge HM. Brief communication: the London atlas of human tooth development and eruption. Am J Phys Anthropol 2010;142: 481-490.
