# Supplementary material for: Leprosy at the edge of Europe—Biomolecular, isotopic and osteoarchaeological findings from medieval Ireland
Source: PLoS One. 2018 Dec 26;13(12):e0209495. doi: 10.1371/journal.pone.0209495 (PMC6306209; doi:10.1371/journal.pone.0209495)
Supplement: S3 File — (DOCX) [file pone.0209495.s003.docx]

**Supporting information S3 file – stable isotopes**


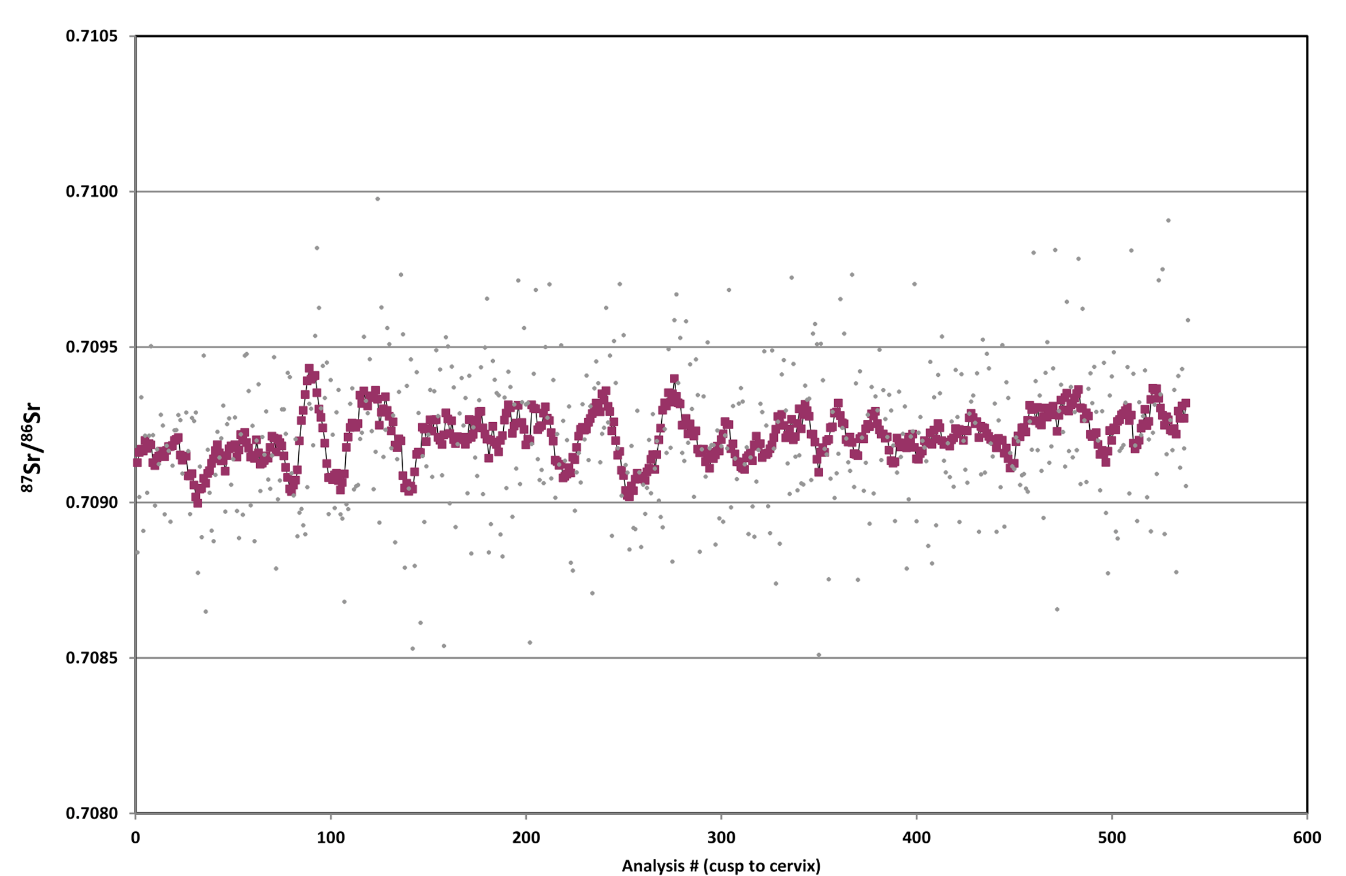


**Figure A3. Strontium isotopic profile of SkCXLVIII from Golden Lane, Dublin.**


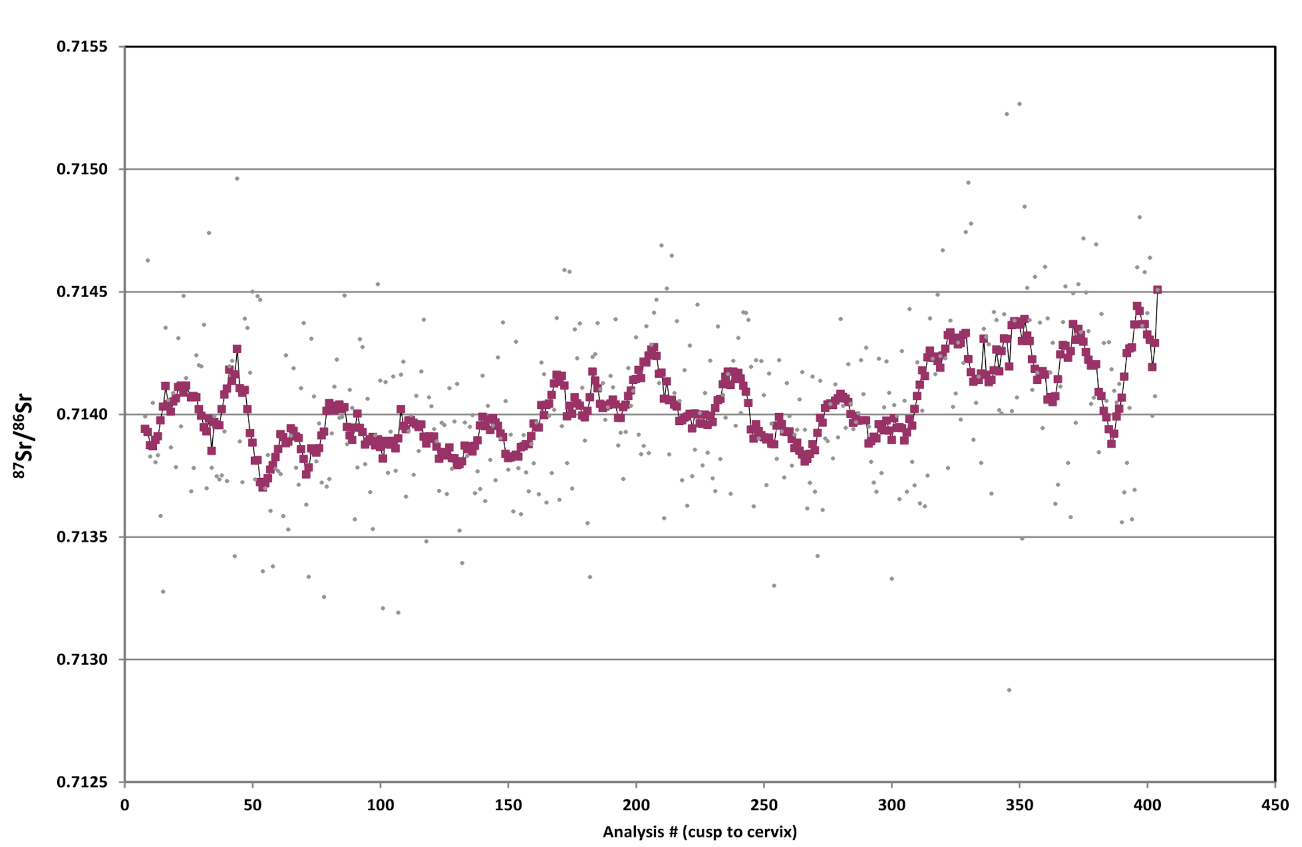


**Figure B3. Strontium isotopic profile of Sk CXCV from Golden Lane, Dublin.**


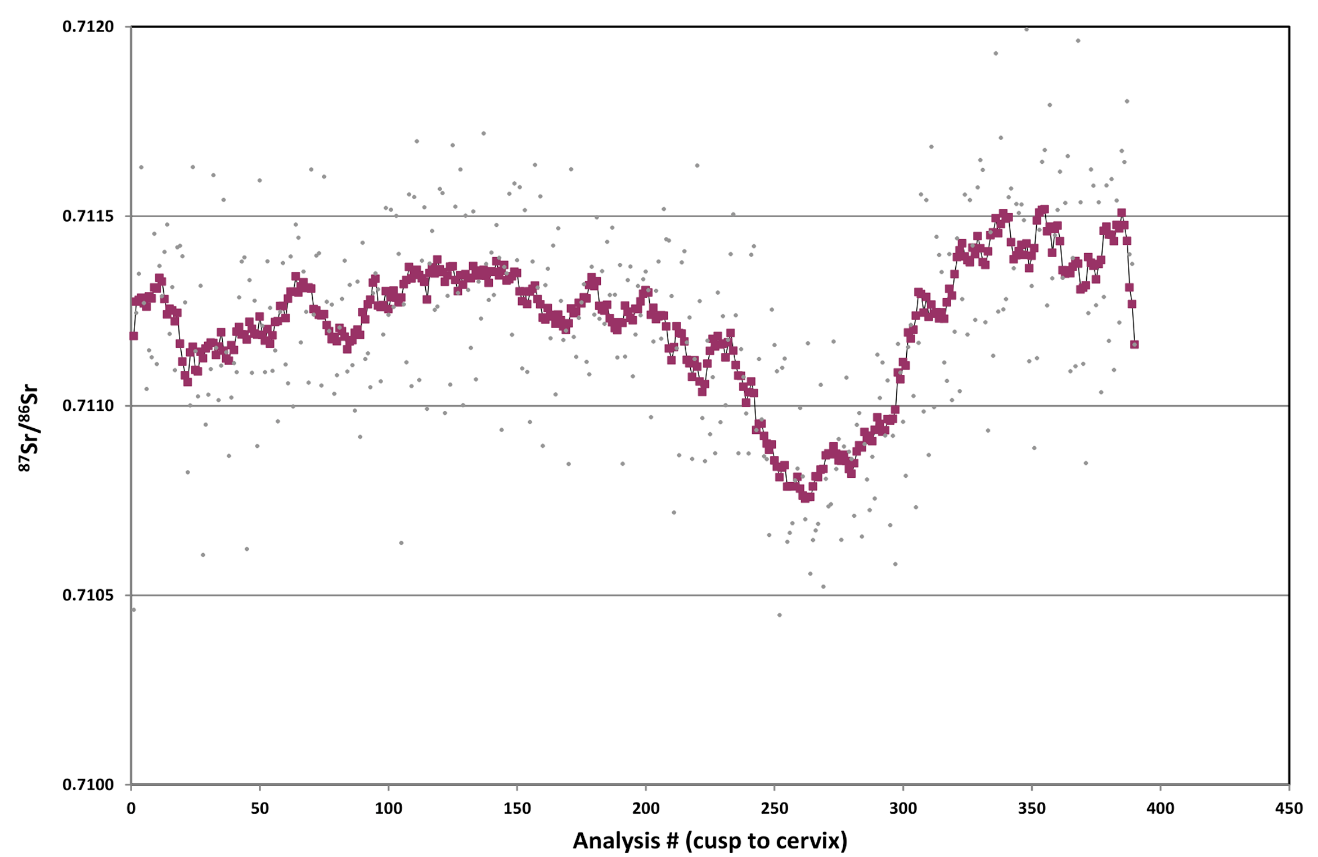


**Figure C3. Strontium isotopic profile of SkCCXXX from Golden Lane, Dublin.**


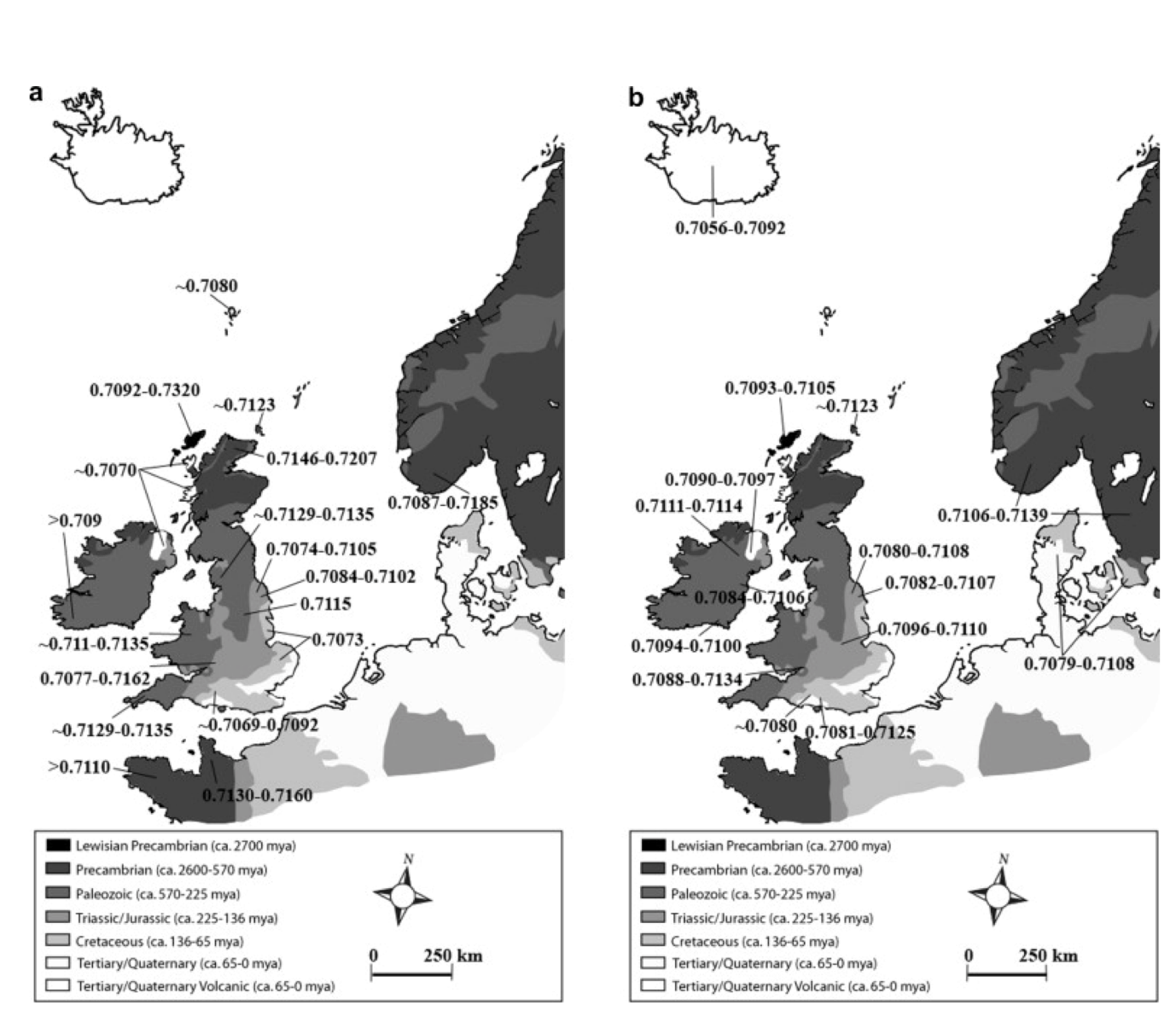


**Figure D3. The ‘map’ of oxygen isotopes in modern spring and well waters. Carbon and oxygen isotopes of structural carbonate (18Oc) in the tooth enamel. For comparison this has been converted to phosphate equivalent values (18Op) using Chenery *et al* (2012) from which drinking water (dw) equivalent values have been calculated according to Longinelli (1984) and Daux *et al* (2008).**

**References**

Chenery CA, Pashley V, Lamb AL, Sloane HJ, Evans JA. The oxygen isotope relationship between the phosphate and structural carbonate fractions of human bioapatite. Rapid Commun Mass Spec 2012;26: 309-319.

Daux V, Lécuyer C, Héran MA, Amiot R, Simon L, Fourel F*,* et al. Oxygen isotope fractionation between human phosphate and water revisited. J Hum Evol 2008;55(6): 1138-1147.

Longinelli A. Oxygen isotopes in mammal bone phosphate: a new tool for paleohydrological and paleoclimatological research? Geochim Cosmochim Acta 1984;48(2): 385-390.
