# Supplementary material for: Leprosy at the edge of Europe—Biomolecular, isotopic and osteoarchaeological findings from medieval Ireland
Source: PLoS One. 2018 Dec 26;13(12):e0209495. doi: 10.1371/journal.pone.0209495 (PMC6306209; doi:10.1371/journal.pone.0209495)
Supplement: S4 File — (DOCX) [file pone.0209495.s004.docx]

**Supporting information S4 file – radiocarbon dating**

**Figure A4. Plot of the radiocarbon dates for the individuals included in the study based on OxCal v4.3.2 (Bronk Ramsey 2017) and r:5 IntCal13 atmospheric curve (Reimer et al. 2013).**

**References**

Bronk Ramsey C. 2017. OxCal v4.3.2 < <https://c14.arch.ox.ac.uk/oxcal.html#program> >

Reimer PJ, Bard E, Bayliss A, Warren Beck J, Blackwell PG, Bronk Ramsey C, et al. IntCal13 and Marine13 radiocarbon age calibration curves 0-50,000 Years cal BP. Radiocarbon 2013;55(4): 1869-1887.
