# Supplementary material for: Leprosy at the edge of Europe—Biomolecular, isotopic and osteoarchaeological findings from medieval Ireland
Source: PLoS One. 2018 Dec 26;13(12):e0209495. doi: 10.1371/journal.pone.0209495 (PMC6306209; doi:10.1371/journal.pone.0209495)
Supplement: S5 File — (DOCX) [file pone.0209495.s005.docx]

**Supporting information S5 file – Table of details of sites and studies referred to in Figure 5.**

| **Symbol No.** | **Burial No.** | **Type** | **Period, century** | **Reference** |
| --- | --- | --- | --- | --- |
| 1 | CCXXX | 2F | 11^th^-13^th^ | Present study |
| 2 | CXCV | 3I | 12^th^-13^th^ | Present study |
| 3 | Sk2 | 3I | 10^th^-12^th^ | Taylor et al, 2013 |
| 4 | Sk7 | 3I | “ | “ |
| 5 | Sk19 | 3I | “ | “ |
| 6 | Sk8 | 2F | 11^th^-12^th^ | “ |
| 7 | Sk14 | 2F | 10-12^th^ | “ |
| 8 | Sk27 | 2F | 11^th^ 12^th^ | Roffey et al, 2017 |
| 9 | G708 | 3 | 10^th^ -12^th^ | Unpublished |
| 10 | 1914 | 3I | 13^th^-16^th^ | Taylor et al, 2009 |
| 11 | GC96 | 3I | 5^th^ – 7^th^ | Inskip et al, 2015 |
| 12 | T439 | 3I | 9^th^-11^th^ | Inskip et al, 2017 |
| 13 | STA 538 | 3I | 14^th^-15^th^ | Donoghue et al, 2017 |
| 14 | STA 549 | 3I | “ | “ |
| 15 | STA 642 | 3I | “ | “ |
| 16 | STA 930 | 3I | “ | “ |
| 17 | G483 | 3 | 13^th^ -16^th^ | Watson & Lockwood, 2009 |
| 18 | Jorgen 625 | 3I | 13^th^-14^th^ | Schuenneman et al, 2013 |
| 19 | Jorgen 722 | 3I | “ | Schuenneman et al, 2018 |
| 20 | Jorgen 507 | 3K | “ | “ |
| 21 | Refshale 16 | 2F | “ | Schuenneman et al, 2013 |
| 22 | Jorgen 404 | 3I | “ | Schuenneman et al, 2018 |
| 23 | Jorgen 427 | 3I | “ | “ |
| 24 | Jorgen 533 | 3I | “ | “ |
| 25 | Jorgen 749 | 2F | “ | “ |
| 26 | 3077 | 2F | 11^th^–12^th^ | Economou et al, 2013 |
| 27 | 3092 | 2F | 10^th^ - 14^th^ | “ |
| 28 | 3093 | 3I | 10^th^-14^th^ | “ |
| 29 | 188 | 3M | 9^th^ | Schuenneman et al, 2018 |
| 30 | 503 | 3M | 10^th^–11^th^ | Monot et al, 2009 |
| 31 | 222 | 3K | 10^th^-11^th^ | “ |
| 32 | KD271 | 3K | 7^th^ | “ |
| 33 | Sk11 | 3K | 7^th^-8^th^ | Schuenneman et al, 2018 |
| 34 | 2A | 3 | 8^th^-9^th^ | Watson & Lockwood, 2009 |
| 35 | 3A | 3 | 8^th^-9^th^ | “ |
| 36 | T18 | 2F | mid-late 7^th^ | Schuenneman et al, 2018 |
| 37 | K2-B116 | 3 | 4^th^-5^th^ | Monot et al, 2009 |
| 38 | KK20/1 | 3K | 8^th^-9^th^ | “ |
| 39 | Burial 5b | 3L | 1^st^-4^th^ | Taylor et al, 2009 |

**References**

Donoghue HD, Johnson J, Masaku O, Huhulski H, Jamil S, Cui A, et al. Aperçus des connaissances sur la présence de *Mycobacterium leprae* en Europe médiévale occidentale, centrale et orientale à partir de l'analyse moléculaire menée sur la population de Saint Thomas d’Aizier (Eure, fin XII^e^–XVI^e^ s.). Groupe Des Paléopathologistes De Langue Francaise. Collogue, Université de Caen, Normandie, 24-25 Mars, 2017.

Economou C, Kjellström A, Lide ́n K, Panagopoulos I. Ancient-DNA reveals an Asian type of *Mycobacterium leprae* in medieval Scandinavia. J Archaeol Sci 2013;40: 465-470. Corrigendum appears in J Archaeol Sci 2013;40: 2867.

Inskip SA, Taylor GM, Zakrewski S, Mays SA, Pike AWG, Llewellyn G, et al. Osteological, biomolecular and geochemical analysis of an early Anglo-Saxon case of lepromatous leprosy. PLOS One 2015;10(5): e0124282.

Inskip SA, Taylor GM, Anderson S, Stewart GR. Leprosy in Pre-Norman Suffolk: Biomolecular and geochemical analysis of the woman from Hoxne. J Med Microbiol 2017;6: 1640-1649. doi: 10.1099/jmm.0.000606.

Monot M, Honoré N, Garnier T, Zidane N, Sherafi D, Paniz-Mondolfi A et al. Phylogeography of Leprosy. Nat. Genet 2009;41**:** 1282-1289. doi:10.1038/ng.477

Roffey S, Tucker K, Filipek-Ogden K, Montgomery J, Cameron J, O’Connell T, Evans J, Marter P, Taylor GM. Investigation of a Medieval pilgrim burial excavated from the leprosarium of St Mary Magdalen, Winchester, UK. PLoS Neglected Tropical Diseases 2017;11: e0005186. doi:10.1371/journal.pntd.0005186

Schuenemann VJ, Singh P, Mendum TA, Krause-Kyora B, Jäger G, et al. Genome-wide comparison of Medieval and modern *Mycobacterium leprae*. Science 2013;341: 179-183.

Schuenneman VJ, Avanzi C, Krause-Kyora B, Seitz A, Herbig A, Inskip S, Bonazzi M et al. Ancient genomes reveal a high diversity of *Mycobacterium leprae* in medieval Europe. PloS Pathog 2018;14(5): e1006997. doi: 10.1371/journal.ppat.1006997.

Taylor GM, Tucker K, Butler R, Pike AWG, Lewis J, Roffey S, et al. Detection and strain typing of ancient *Mycobacterium leprae* from a Medieval leprosy hospital. PLoS ONE 2013;8(4): e62406. doi: 10.1371/journal.pone.0062406.

Taylor GM, Blau S, Mays SA, Monot M, Lee OY-C, Minnikin DE, et al. *Mycobacterium leprae* genotype amplified from an archaeological case of Lepromatous Leprosy in Central Asia. J Archaeol Sci 2009;36: 2408-2414.

doi:10.1016/j.jas.2009.06.026.

Watson CL, Lockwood DNJ. Single nucleotide polymorphism analysis of European archaeological *M. leprae* DNA. PLoS ONE 2009;4: e7547.
